# Computational Prediction of Neutralization Epitopes Targeted by Human Anti-V3 HIV Monoclonal Antibodies

**DOI:** 10.1371/journal.pone.0089987

**Published:** 2014-02-25

**Authors:** Evgeny Shmelkov, Chavdar Krachmarov, Arsen V. Grigoryan, Abraham Pinter, Alexander Statnikov, Timothy Cardozo

**Affiliations:** 1 Department of Biochemistry and Molecular Pharmacology, New York University School of Medicine, New York, New York, United States of America; 2 Public Health Research Institute Center, Rutgers New Jersey Medical School, Newark, New Jersey, United States of America; 3 Center for Health Informatics and Bioinformatics, New York University School of Medicine, New York, New York, United States of America; Shanghai Medical College, Fudan University, China

## Abstract

The extreme diversity of HIV-1 strains presents a formidable challenge for HIV-1 vaccine design. Although antibodies (Abs) can neutralize HIV-1 and potentially protect against infection, antibodies that target the immunogenic viral surface protein gp120 have widely variable and poorly predictable cross-strain reactivity. Here, we developed a novel computational approach, the Method of Dynamic Epitopes, for identification of neutralization epitopes targeted by anti-HIV-1 monoclonal antibodies (mAbs). Our data demonstrate that this approach, based purely on calculated energetics and 3D structural information, accurately predicts the presence of neutralization epitopes targeted by V3-specific mAbs 2219 and 447-52D in any HIV-1 strain. The method was used to calculate the range of conservation of these specific epitopes across all circulating HIV-1 viruses. Accurately identifying an Ab-targeted neutralization epitope in a virus by computational means enables easy prediction of the breadth of reactivity of specific mAbs across the diversity of thousands of different circulating HIV-1 variants and facilitates rational design and selection of immunogens mimicking specific mAb-targeted epitopes in a multivalent HIV-1 vaccine. The defined epitopes can also be used for the purpose of epitope-specific analyses of breakthrough sequences recorded in vaccine clinical trials. Thus, our study is a prototype for a valuable tool for rational HIV-1 vaccine design.

## Introduction

The HIV-1 virus causes AIDS, which is a global and devastating public health problem. The development of a vaccine that could protect humans from HIV-1 infection is therefore a high public priority [1], [2], [3]. However, several factors, most notably the extraordinary antigenic diversity of HIV-1 strains, have obstructed the development of such a vaccine [1]. In particular, many known neutralizing antibodies are type-specific or narrowly cross-reactive and cannot neutralize the vast majority of circulating HIV-1 strains. This phenomenon appears to be due to the molecular heterogeneity present on the HIV-1 viral surface.

The gp120 glycoprotein of HIV-1 is the major surface-exposed viral target for anti-HIV-1 Abs [4], [5]. It consists of a relatively sequence-conserved core and five sequence-variable (V1–V5) loops, which are further covered by a glycan shield [6], [7]. The gp120 variable loops, although among the few regions that consistently elicit Abs in the host, have been generally considered as unlikely vaccine targets for cross-strain protective Abs as their sequences differ from strain to strain [8]. However, recent studies have provided extensive evidence of structural conservation hidden within these sequence-variable loops [9] and demonstrated that a significant level of immunologic cross-reactivity is present among V2 and V3 loops of diverse HIV-1 strains [10], [11]. Importantly, the first epitopes ever associated with protection from HIV-1 acquisition were found to reside in the V2 loop [12]. The antigenic heterogeneity of gp120 is therefore best described as “rare epitopes targeted by protective Abs occurring variably across circulating strains and hidden among numerous decoy epitopes”. Accordingly, precisely identifying specific epitopes targeted by mAbs is both an experimental and computational challenge.

The presence of an epitope in an HIV-1 strain can be experimentally estimated by *in vitro* neutralization of the HIV-1 strain by a query Ab. However, scaling this approach up via high-throughput means to assess the thousands of circulating worldwide HIV-1 variants, the population of which are also constantly evolving, would not be feasible. The only computational alternative proposed to date is a heuristic Signature Motif Method (SMM) [13], [14]. This approach represents a neutralization epitope with a sequence pattern (signature motif). The presence of the signature motif in the viral sequence suggests the presence of the corresponding neutralization epitope on the gp120 of the virus. SMM allows quick prediction of the presence of Ab neutralization epitopes in any number of HIV-1 strains solely from the viral gp120 amino acid sequence. Such a quick prediction enabled several useful analyses [13], [14], [15], [16], [17] that would not be feasible by experimental means.

Unfortunately, the accuracy of SMM is far from ideal. The limited accuracy is the result of the imperfect epitope-defining approach, which only considers the side chain composition of an epitope but not the conformational preferences of its peptide backbone [13], [14]. Suboptimal accuracy could limit the potential utility of SMM in analyzing circulating viruses or viral isolates from clinical trials, as well as narrow the applicability of the method in vaccine immunogen design. Accordingly, in this study we developed a more accurate high throughput computational method, the Method of Dynamic Epitopes (MDE), which predicts dynamic (both in sequence and in structure) epitopes of human anti-HIV-1 Abs. We optimized our method for two different anti-V3-loop monoclonal antibodies (mAbs), 2219 [18] and 447-52D [19], and used this method in one of its useful applications: to obtain new calculated measurements of breadth of reactivity of these mAbs among major prevalent HIV-1 group M subtypes and circulating recombinant forms (CRFs).

## Results

### Optimization of the Method of Dynamic Epitopes

**Figure 1:**
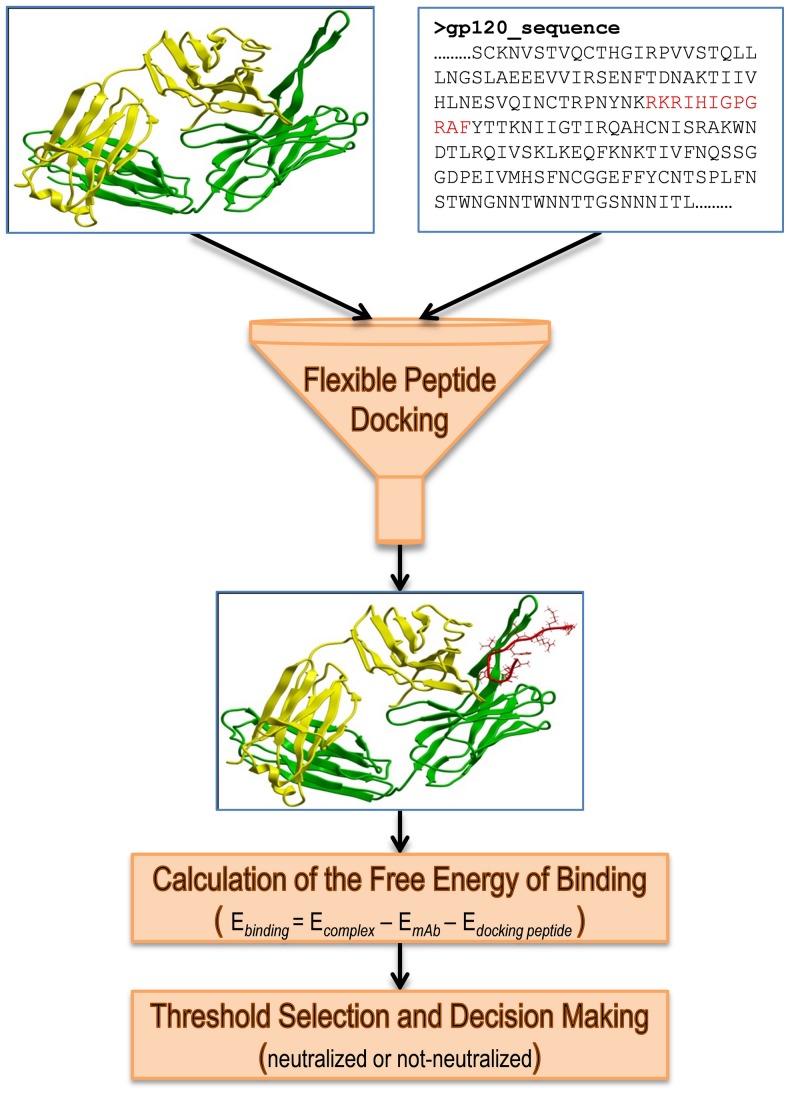
Scheme of the Method of Dynamic Epitopes (MDE). In the first step, a docking peptide (shown in red) is selected from an epitope-containing region of gp120 sequence. Then, the optimal conformation of this docking peptide on the surface of a mAb in question is found using the Flexible Peptide Docking procedure. Next, the predicted free energy of binding of the docked peptide conformation to the mAb is calculated. The prediction of whether the neutralization epitope of the tested mAb is present in the strain is made based on the value of predicted binding energy and selected energy threshold.

In order to incorporate both amino acid composition and peptide conformation into epitope identification, we used *de novo in silico* docking of a flexible peptide (or Flexible Peptide Docking, FPD), which represents the epitope-containing peptide fragment of HIV-1 gp120 (docking peptide) within the environment of a static 3D structure of the antigen-combining site of specific mAbs (Figure 1). The prediction of whether neutralization could occur between the pair in the absence of epitope masking was made based on attainment of a specific binding energy threshold observed in docking of the viral peptide to the mAb (Figure 1, Text S1). Specifically, the preferred orientation of the peptide obtained in FPD to the mAb corresponds to the most energetically favorable conformation of the complex and, thus, is characterized by the lowest predicted free energy of binding. This predicted binding energy was calculated as E_binding_ = E_complex_ − E_mAb_ − E_docking peptide_, where E_complex_ is a predicted free energy of a complex of a peptide docked into the mAb, while E_mAb_ and E_docking peptide_ are predicted free energies of the mAb and the corresponding conformation of a peptide separately (Figure 1). In other words, this predicted binding energy characterizes a difference between bound and unbound states of a peptide and a mAb, and is a theoretical estimate of a change in the Gibbs free energy upon peptide-mAb binding. Thus, low predicted free energy of binding theoretically corresponds to a high structural complementarity of the assayed peptide to the antigen-combining site of the query mAb and, therefore, a high probability of the existence in the virus’ gp120 of the specific neutralization epitope targeted by the mAb. The energies were calculated from existing structural data on anti-V3 loop HIV-1 neutralizing mAbs, and the method for classifying peptides/viruses into those bearing the neutralization epitope and those missing the epitope was optimized in the space of variable parameters, such as peptide length, using experimental data. In order to develop and optimize this method, we needed training data sets consisting of crystallographic peptide:mAb complexes and matched viral neutralization measurements.

#### Structural data on anti-V3 mAbs

To date, co-crystal structures of several gp120-targeted mAbs have been published in a complex with their gp120-derived antigens [20], [21], [22], [23], [24], [25]. For our study, we selected mAbs 2219 (with three available crystallographic conformations) and 447-52D (with six available crystallographic conformations), which are specific for the V3 loop of the gp120 (see Table S1). The rationale for this choice is that (a) the V3 loop serves as a thoroughly studied prototype in terms of variable loop antigenic variation, within which virus neutralization epitopes lie, (b) the effects of epitope masking in the V3 loop can be controlled [15], [26]; (c) our recent study suggests that immunogens that elicit 2219-like Abs are potentially protective as vaccine components [16].

#### Experimental neutralization data set

To optimize the method, we used a panel of 59 chimeric pseudoviruses (psVs) containing diverse V3 sequences presented in a common gp120 backbone in which each V3 domain is equally highly exposed (unmasked) [27]. Thus, loss or gain of neutralization of these psV by anti-V3 mAbs as a result of V3 sequence changes can only be due to changes in the epitope and not due to masking or other long-range effects. All psVs were experimentally tested for their ability to be neutralized by mAbs 2219 and 447-52D (Methods and Figure S1). We considered that neutralization of a given psV had not occurred if 50% neutralization of this psV was never achieved in the range of concentrations tested in the experiment (i.e., IC50>20 µg/ml). Otherwise we considered a given psV to be neutralized detectably (Text S1).

#### Optimization

In order to optimize MDE for prediction of the presence of 2219 and 447-52D mAb neutralization epitopes in any V3 loop sequence, we sought to identify an optimal docking model for each of these mAbs. For a given mAb, the optimal docking model is the model that allows the best classification of our set of 59 unmasked psVs by this mAb into “neutralized” and “not neutralized” based on the binding energy score observed in FPD. Each docking model for each mAb has three independent variables, the space of which needs to be searched for the optimal model. One variable is the conformation (or a set of different conformations; Text S1) of the mAb that is used for the FPD. While multiple conformations of the same mAb crystallized in complex with different V3 peptides vary only slightly in their structure (as the V3 loop has a relatively conserved ß-hairpin structure), we found that these conformations still result in substantially different quality of prediction of neutralization of our psVs set since the determinants of binding are highly precise (Figures S2 and S3). Similarly, the approach is highly dependent on the selection of boundaries of the docking peptide for each mAb (Figures S2 and S3). Therefore, two other variables of a docking model are the coordinates of the docking peptide in the V3 sequence, i.e. the starting and ending positions of the V3 segment used in the docking. We analyzed 315 docking models for mAb 2219 and 630 docking models for mAb 447-52D, and identified the optimal docking models for each mAb as described in the Methods section. For the mAb 2219, the optimal mAb conformation is the conformation 2B0S (Table S1) and the optimal starting and ending positions of a docking peptide are the positions 10 and 13, respectively (Figure S2). On the other hand, for 447-52D the optimal mAb conformation is the 1Q1Jq (Table S1), and the optimal starting and ending positions are 9 and 20 (Figure S3). The area under the receiver operating characteristic curve (AUC) for 2219 was estimated to be equal to 0.93, 95% confidence interval (CI) equal to (0.86; 0.99), while for 447-52D it is 0.85, 95% CI (0.75; 0.95). This performance represents an average gain in accuracy of 11% compared to SMM, the only prior method reported for identifying HIV-1 neutralization epitopes solely based on sequence. The gain in accuracy for MDE over SMM is statistically significant (McNemar’s test p = 0.0073; see Methods and Text S1; Table S2). Furthermore, this performance improvement suggests that the backbone conformational preference (e.g., for a ß-strand) contributes significantly to the formation of the epitope, since MDE incorporates this information and SMM does not.

Genuine high correlations between docking scores and independent *in vitro* or cellular measurements are rare, as highlighted by the long-time pursuit of accurate docking-based binding affinity predictions [28]. The near-perfect correlation of a score generated solely from *in silico* data (the peptide sequence and the mAb atomic coordinates) with independent *in vitro* measurements of neutralization for psV and mAb suggests that the docking calculations, the 2219 structure 2B0S [25], and the neutralization measurements in the experimental data set are all accurate. In addition, as we are using an unconstrained peptide to represent a segment of the V3 loop that is normally connected at its loop ends, this result further demonstrates that the crown of the V3 loop, which is located at the tip of this long loop, is almost completely unconstrained by the loop stems in the biological reality of our SF162-based chimeric psVs and folds as if it was an unconstrained free peptide, which can therefore be modeled by docking of only the crown fragment.

### Estimation of mAb Epitope Conservation across Circulating HIV-1 Subtypes

**Figure 2:**
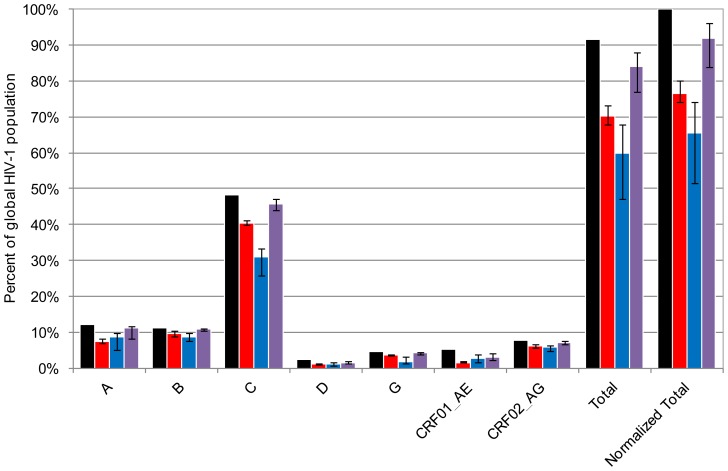
Graphical representation of estimates of epitope conservation of mAbs 2219 and 447-52D. Black bars represent prevalence of HIV-1 subtypes/CRFs among global HIV-1 population (data from Hemelaar, et al., 2011). For example, the height of the black bar labelled as ‘A’ represents prevalence of subtype A among the global HIV-1 population (12.03%). Other bars represent proportions of HIV-1 population of each clade that exhibit neutralization epitopes of the mAb 2219 (red), 447-52D (blue), or at least one of these mAbs (violet). These data are also summarized in Table 2.

To demonstrate one important application of MDE, we used it to determine the conservation of epitopes targeted by mAbs 2219 and 447-52D across major circulating worldwide HIV-1 subtypes and CRFs. Specifically, we considered five subtypes (A, B, C, D, G), and two CRFs (CRF01_AE and CRF02_AG), as each of them is responsible for at least 1% of worldwide infections [29]. Obtained estimates for epitope conservation are summarized in the Table 1. These estimates answer the question: what percentage of circulating HIV-1 viruses bear the neutralization epitope targeted by mAb 2219 and by mAb 447-52D. Figure 2 and Table 2 show the results of projecting these numbers onto the global estimates of worldwide prevalence of different HIV-1 subtypes and CRFs.

**Table 1:** Percentage of HIV-1 strains of different clades that contains epitopes of mAbs 2219 and 447-52D.

mAb	A	B	C	D	G	CRF01_AE	CRF02_AG
2219	62.4	85.5	83.5	46.5	82.1	31.6	78.4
447	72.7	76.8	64.2	52.0	39.8	50.8	74.7
2219 or447	92.5	97.1	95.1	65.3	90.3	60.2	93.0

Note: Column headings display subtype/CRF names. Cells show the percentage of HIV-1 population of a given subtype/CRF that contains epitopes of the indicated mAbs.

**Table 2:** Percentage of HIV-1 strains circulating worldwide that could be neutralized by mAbs 2219 and 447-52D.

mAb	A (12.0%)	B (11.3%)	C (48.2%)	D (2.5%)	G (4.6%)	CRF01_AE(5.1%)	CRF02_AG(7.7%)	Total(91.5%)	Normalizedtotal (100%)	Global effectivebreadth range (in %)
2219	7.5	9.7	40.3	1.2	3.8	1.6	6.1	70.1	76.6	6.9–27.0
447	8.7	8.7	31.0	1.3	1.8	2.6	5.8	59.9	65.5	6.6–26.8
2219 or 447	11.1	11.0	45.8	1.6	4.2	3.1	7.2	84.0	91.8	9.1–36.2

Note: Each clade-specific column heading displays a subtype/CRF name and prevalence of this subtype/CRF (i.e., percentage) among worldwide circulating viruses. Clade-specific cells show percentages of the global HIV-1 population, all of that clade, that contain the epitopes of the indicated mAbs. For example, 7.5% for 2219 and subtype A indicates 7.5% of the global HIV-1 population, which is 62.4% (see Table 1) of viruses of the subtype A. The ‘Global effective breadth range’ column shows the estimated minimal and maximal percentage of circulating HIV-1 viruses of the tested clades that are predicted to be neutralized by the indicated Ab (i.e. % of viruses exhibiting unmasked antibody epitopes).

The clade-specific estimates for 2219 are generally consistent with the previously published assessment based on SMM [14]. Minor variations occurred due to the improvement in methodology (usage of the more precise MDE versus SMM) and due to changes in HIV-1 global distribution between 2004 and 2007 [29], [30]. On the contrary, the estimates for 447-52D are substantially different between the current and the previous study. Specifically, 447-52D was previously estimated to be highly subtype B specific [14] and targeted only against 11% of global strains, while our new data suggests that it can theoretically target up to 65.5% of globally circulating HIV-1 (Table 2). The main difference between these numbers is in the estimate of coverage of the most prevalent worldwide subtype C: 64.2% here versus 0% in Swetnam, et al. This dissimilarity appears to be due to the previously reported [15] imperfection of an epitope signature motif for 447-52D derived via SMM. Briefly, according to SMM, a virus is required to have arginine at the position 18 of the V3 loop to be neutralized by 447-52D. R18 is almost exclusive for the subtype B in contrast to the subtype C where Q18 is common. Therefore, SMM detected close to zero coverage of the subtype C by this mAb [14]. However, it was recently found that strains with Q18 could also be neutralized by 447-52D [10], [15], [21]. These data are consistent with our current results and support the conclusion of our current study that 447-52D epitope conservation was previously highly underestimated. Nevertheless, it is important to highlight that the position 18 of the V3 loop distinguishes between high affinity (R18 containing) and low affinity (Q18 containing) versions of the 447-52D epitope [21]. The low affinity binding might be sufficient to allow neutralization only in strains with extremely low masking (e.g. SF162), but not in primary isolates.

Another intriguing result is the estimate of the coverage of the circulating HIV-1 population by 2219 and 447-52D together (Figure 2, Tables 1 and 2). Our data suggest that the dynamic neutralization epitopes for at least one of the two mAbs are conserved (but may be or may not be accessible) in up to 92% of the global HIV-1 population of circulating viruses and more than 95% of all subtype B and C strains.

### Estimation of Effective Breadth of Reactivity of the Anti-V3 mAbs

The presence of an epitope is not sufficient for neutralization due to the effects of epitope masking (i.e. evolutionary favorable ‘hiding’ of an epitope by other parts of gp120) [15]. To address this, we used MDE to obtain the improved estimates for masking of epitopes 2219 and 447-52D (see Methods, Text S1, and Figure S4). Previous estimates [15] were made based on SMM, and thus could be less precise.

Only two HIV-1 subtypes (B and C) were well represented in the experimental dataset (Table S3). Our data suggest that the 2219 epitope is unmasked in 50% and 5% of strains of these subtypes respectively, resulting in the estimated subtype specific neutralization breadth of mAb 2219 of 42.7% for the subtype B and 4.2% for the subtype C (that in sum represent 6.9% of all circulating HIV-1 strains). On the other hand, the 447-52D epitope is unmasked in 46.4% of the subtype B and 8.3% of the subtype C strains. The subtype specific neutralization breadth of mAb 447-52D, therefore, is equal to 35.6% for the subtype B and 5.4% for the subtype C (6.6% of all circulating HIV-1 strains). Similarly, at least one of these two epitopes is estimated to be unmasked in 56.2% of subtype B and 6.2% subtype C strains. Therefore, if elicited simultaneously, these mAbs are predicted to neutralize 54.6% of the subtype B and 5.9% of the subtype C strains (9.1% of all circulating HIV-1 strains).

The similar estimation of clade-specific masking and clade-specific neutralization breadth, however, cannot be performed for the other subtypes and CRFs (A, D, G, CRF01_AE, and CRF02_AG) due to the small sample size (Table S3). Therefore, here we report the global effective neutralization breadth of each mAb across all the considered subtypes/CRFs as a range limited by minimal and maximal possible values for masking in these outlier clades (Table 2). The minimal value was calculated as a percentage of all HIV-1 strains of the considered clades that can be neutralized by a given mAb assuming that an epitope targeted by this antibody is always masked in all A, D, G, CRF01_AE, and CRF02_AG strains. In contrast, the maximal value is the percentage of all HIV-1 strains of the clades that can be potentially neutralized by a given mAb assuming that an epitope targeted by this antibody is always accessible/unmasked in the A, D, G, CRF01_AE, and CRF02_AG.

Our final estimates (Table 2) suggest that the potential neutralization ranges of mAbs 2219 and 447-52D are very similar: (6.9%–27.0%) for 2219 versus (6.6%–26.8%) for 447-52D. On the other hand, the simultaneous elicitation of these mAbs would increase coverage of the worldwide HIV-1 population (9.1%–36.2%).

Although the extent of masking of 2219 and 447-52D epitopes in A, D, G, CRF01_AE and CRF02_AG could not be accurately estimated using the available data, it is qualitatively very unlikely that the vast majority of the viruses of those clades are unmasked to the 2219 and 447-52D epitopes. On the other hand, it is also unlikely that all of them are masked.

Therefore, while only the theoretical maximal and minimal bounds may be calculated here with certainty, a rule of thumb estimate for the percentage masked in these clades might be the same as masking percentage in subtype C (Table S3). This assumption would result in the overall breadth estimate of mAbs 2219 and 447-52D of 7.9% and 8.3% respectively. Similarly, in case of the simultaneous elicitation of these mAbs, 10.8% of all circulating strains could be neutralized.

## Discussion

We have described a new method, MDE, that accurately predicts the presence of neutralization epitopes in the V3 loop of any HIV-1 virus solely from the viral sequence and the crystallographic structure of the anti-V3 mAbs 2219 and 447-52D. The method accounts for both the side chain composition of the epitope and for the conformational preferences of the peptide backbone on which the epitope is located, predicting fully dynamic HIV-1 epitopes. The improved accuracy of the procedure over SMM (Table S2) promises greater utility in all the applications of computational epitope prediction. In particular, more reliable estimates of neutralization epitope occurrence across circulating HIV-1 strains and neutralization ranges of individual mAbs (the latter is dependent on epitope masking estimates) were calculated. Although we only tested two mAbs, 2219 and 447-52D, given the fact that they have completely different V3 loop-binding modes, we anticipate that our approach can be extended to other mAbs with known structures. Thus, the method could be ideal for evaluating epitopes in HIV-1 variable loops.

The major utility of this method is to provide an additional objective measure encompassing all of the sequences of circulating group M HIV-1 viruses for use in determining rational combinations of immunogens in a vaccine. The reverse vaccinology method, in which an immunogen is designed to mimic and preferentially or exclusively display the epitope targeted by a single mAb, is the most promising pathway to translate the activity of broadly neutralizing mAbs into vaccine immunogens. Immunogens produced by the reverse vaccinology method may be combined into a multivalent vaccine, but in the absence of estimates like those we have generated, two immunogens may overlap substantially and target the same viral population, reducing vaccine efficacy and leave many viruses untargeted. Our method yields the exact sets of viruses that bear the target epitopes, allowing combinations that minimize overlap and maximize the breadth of coverage of circulating viruses. The method may also be used, as SMM was [16], for analysis of correlations between specific breakthrough viral epitopes and vaccine efficacy in vaccine clinical trials (i.e. sieve analysis) [31].

Our study also illustrates the danger of using molecular docking without rigorous optimization and performance evaluation similar to the one we employed here. As described above, the AUC of a docking-based prediction method can range from 0.9 to as low as 0.5 if the method is not optimized (Figures S2 and S3). However, if the optimal docking model is found explicitly based on a training set of neutralization measurements, the AUC can exceed 0.9.

One limitation of our study is that the effects of conformational changes due to mutations elsewhere in gp120 (except for the crown region of the V3 loop) were not evaluated. Another limitation is that the estimation of the extent of masking of the mAb epitopes described here is purely empirical, and is based on a relatively small sample of isolates with highly underrepresented clades A, D, G, CRF01_AE, and CRF02_AG (primarily containing subtype B and C strains). While overcoming the former limitation would lead to a formidable computational problem from the technical point of view (molecular modeling of the whole gp120 containing thousands of atoms would require unrealistic computational resources), the latter could potentially be addressed in the future as more data on masking of epitopes in gp120 become available. The availability of the recently published crystallographic structure of a soluble cleaved HIV-1 envelope trimer [7] provides only a limited amount of additional structural information relevant to masking as it depicts only one strain of HIV-1, while masking of the V3 may be very different mechanistically from strain to strain.

Various broadly neutralizing mAbs against the HIV-1 CD4 binding site, the V2 and V3 loops, and the gp41 membrane proximal region, some of which target more complicated discontinuous, glycopeptide, or lipid-peptide epitopes are known [11], [32], [33], [34]. The components necessary to predict the presence of an epitope from sequence alone are a conformational search and an energy calculation to establish the presence of the epitope and, separately, precise estimates of masking of the epitope and long-range interactions influencing the epitope. In this study, we have established the maximally accurate conformational search and energy calculation protocol for the ideal V3 loop system, in which a linear peptide containing the epitope recapitulates to a large degree the unmasked epitope within the pseudovirus. The main obstacle to accurate prediction of neutralization breadth in this report is the masking estimate. Conversely, the path towards applying MDE to more complex epitopes may be easy in some cases and difficult in others. In principle, our method, which can also dock a glycopeptide or lipidated peptide using the same conformational search and energy evaluation protocol, can be adapted for these mAbs without significant modification. This method, therefore, may be useful for evaluating the neutralization breadth of many anti-HIV-1 mAbs and for calculating rational combinations of these Abs for vaccine design. However, adaptation of the method for discontinuous epitopes would require highly accurate protein-protein docking or full folding simulations of entire gp120 molecules, which is likely beyond the reach of current conformational search methods.

The only known molecular correlate of risk for HIV-1 infection was recently found to be a region in the V1V2 loops of gp120: high titers of plasma anti-V1V2 Abs were shown to be associated with a decreased risk of HIV-1 infection in RV-144 vaccine recipients [3], [12]. An antibody-targeted epitope that is associated with protection from HIV-1 infection is likely present in this region. Vaccines designed to target this region face the identical problem of antigenic variation addressed in this paper. As neutralizing mAbs targeting this region are reported, our tool for straightforward prediction of epitopes of these mAbs across circulating HIV-1 strains may be a critical asset in translating the properties of those mAbs into a matching, protective anti-HIV-1 vaccine immunogen cocktail.

The most important application of our method is the prediction of the effective neutralization breadth of anti-gp120 mAbs *in silico*. Such estimates allow the molecular epidemiologic modeling of important lead immunogens in HIV-1 vaccine development.

Our method can also be, in principle, applied to other high profile public health cases of antigenic variation such as influenza. Finally, expansion of the list of known Ab epitopes using our computational protocol can greatly facilitate the development of novel preventive vaccines as well as novel immunotherapeutic approaches for many infectious pathogens.

## Methods

### Structural Data

Conformations of mAbs 2219 and 447-52D were derived from co-crystal structures of the three V3∶2219 [25] and six V3∶447-52D [21], [22], [24] complexes respectively (Table S1).

### Experimental Pseudovirus (psV) Neutralization Data

Chimeric psVs were constructed by the replacement of the original V3 loop of HIV-1 strain SF162 env with one of 59 different V3 loop sequences (Figure S1). Thus, these psVs vary only at residues in the V3 region (primarily in the V3 crown) which is always unmasked/accessible in SF162 env [26]. The neutralization by 2219 and 447-52D of each of these psVs was assessed with a single-cycle infectivity luciferase assay as described elsewhere [13], [35]. The concentrations for 50% neutralization (IC50) were quantified for each mAb:psV pair. A rationale for dividing the experimental set of psVs into two subsets, those that are neutralized by a given mAb *in vitro* and those that are not, is described in the Text S1.

### Flexible Peptide Docking Procedure

The flexible peptide docking (FPD) was performed using the biased probability Monte-Carlo (BPMC) algorithm of the Molsoft ICM-Pro (http://www.molsoft.com/icm/ph30.html#peptide-docking) [36], [37]. In FPD, the docking peptide with uncharged N- and C-terminal ends was conformationally unconstrained – free to adopt any overall conformation, although bond angles and bond lengths were held fixed. The crystallographic conformation of a mAb was approximated with grid maps. For each docking run, predicted binding energy was evaluated by taking into account the following energy terms: van der Waals (referred as “vw” and “14” in ICM-Pro) [38], hydrogen bonding (“hb”) [38], electrostatics (“el”) [38], dihedral angle deformation (“to”) [38], and entropy (“en”) [36]. To aid the procedure of finding biologically relevant pockets and peptide conformations, the 3D-space of search was biased towards the vicinity of Complementarity Determining Regions (CDR) of a mAb by drawing a box around this region and imposing an energy penalty outside the box. To avoid FPD getting stuck in local minima, multiple start simulations were used and the lowest predicted energy was recorded. The ability of our FPD procedure to find appropriate conformations was also tested in self- and cross-docking experiments of V3 peptides previously crystallized in complex with mAbs 2219 and 447-52D (Text S1, Figures S5 and S6, Structure S1).

### MDE Optimization

Each docking model is set by three docking parameters: mAb conformation, starting and ending positions of a docking peptide. Three conformations were available for 2219 and six for 447-52D (Table S1). Both, single conformation docking models as well as multiple conformation models (i.e., models incorporating multiple conformations of the same mAb) were evaluated in this study (Text S1). The number of tested starting and ending positions was restricted by the available psVs neutralization data and by the structural data. Specifically, a minimal linear epitope of each mAb was mapped on the V3 loop using the available psVs neutralization data: the minimal linear epitope was defined as the shortest linear fragment of the V3 loop, responsible for the unambiguous recognition of the viral envelope by a mAb in question. A starting position of a docking peptide could not exceed that of the experimentally defined minimal linear epitope, while an ending position of a docking peptide could not be less than that of the experimentally defined minimal linear epitope. For 2219, the shortest linear epitope was restricted to the sequence of 4 amino acids covering the V3 region from position 10 to position 13. For 447-52D the minimal linear epitope spans positions 9 through 19. On the other hand, to reduce the search space the maximal length of a docking peptide was restricted to 12 amino acids for 2219 and 14 amino acids for 447-52D (the region of V3 loop localized in immediate vicinity to mAb surface in the available crystal structures [21], [22], [24], [25]).

### Statistical Analysis

The experimental panel of 59 psVs was used for the optimization of the method for each of the two mAbs. Selection of the best model and estimation of its AUC [39], [40] was performed by hold-out validation (with 2/3 training data and 1/3 testing data) repeated 10,000 times with different splits into training and testing data [41]. The 95% confidence intervals of AUC were computed using a normal approximation based on U-statistic theory [42].

Quality of the prediction of the Method of Dynamic Epitopes and the Signature Motif Method was compared using the McNemar’s test. The null hypothesis was that there is no difference in the quality of prediction of the two methods. The null hypothesis was rejected at the alpha level of 0.05.

### Determination of Epitope Conservation of mAbs

Clade-specific epitope conservation of the mAbs was estimated based on the analysis of the distribution of these epitopes in the HIV-1 sequence data stored in the Los Alamos National Laboratory HIV Database (http://www.hiv.lanl.gov; accessed on 9/22/2011). The analysis was similar to a previously published bioinformatics approach [14], however, it was adapted for MDE instead of SMM (Text S1). Also the most recent World Health Organization data on HIV-1 global prevalence were used [29].

### Determination of Epitope Masking Effects and Effective Breadths of Reactivity of mAbs

Estimation of the extent of masking effects in the V3 loop was performed as described elsewhere [15]: an epitope targeted by a given mAbs was classified as ‘masked’ if this epitope was present in a virus, but the *in vitro* neutralization assay [10] showed no detectable neutralization of that same virus by the cognate mAb. The following important methodological adjustments were, however, made in the original protocol. First, a prediction whether a viral strain from a set of previously published experimental HIV-1 neutralization data [10] contains an epitope of a given mAb was made using MDE instead of SMM. As MDE assays for continuous dynamic epitopes, epitopes in strains with unidentified amino acids in positions covered by an optimal docking peptide of the mAbs could not be predicted. Therefore, these strains were filtered out from the analysis. Thus, the analysis for masking effects was performed on 77 strains for the epitope of 2219, and on 53 strains for both, 447-52D, and 2219 and 447-52D together. Second, the estimates of masking effects were calculated separately for each analyzed HIV-1 clade (Table S3). Accordingly, the effective breadth of reactivity of the tested mAbs was then calculated using these clade-specific estimates of masking, instead of the average estimates across all clades utilized by Agarwal, et al.

## Supporting Information

Figure S1 **Neutralization of psVs containing various V3 loop sequences by mAbs 2219 and 447-52D.** **(a)** Sequences of V3 loop and IC_50_ values (in µg/ml) of neutralization by each mAb are shown for 59 SF162 psVs. Amino acids of a V3 loop different from the consensus B sequence are shown in red. The V3 loop sequence is numbered according to the standard V3 numbering described elsewhere [14]. The IC_50_ value denoted as ‘>20’ represents a non-detectable level of neutralization at the range of concentrations used in the experiment. The IC_50_ data labeled with ‘*’ were derived from the previously published study [13]. The IC_50_ cells are colored according to its value from red to blue, where red background corresponds to high neutralization (small IC_50_ values) and blue to low neutralization (large IC_50_ values; see the color code legend in the bottom of the panel); **(b)** Normalized (to 100%) histogram of all IC_50_ values of neutralization from panel (a). The distribution has two distinct populations at concentrations <1 µg/ml and at the concentrations >20µg/ml.
(PDF)

Figure S2 **Illustration of MDE performance for mAb 2219 in the space of all single- and multiple-conformation docking models.** **(a)** prediction AUC values for all tested docking models of mAb 2219 calculated on the whole set of 59 psVs; **(b)** standard errors of prediction AUC values for corresponding tested docking models of mAb 2219. For both panels, ‘Start’ and ‘End’ are starting and ending positions of tested docking peptides; mAb conformation IDs correspond to the crystal structures in Table S1; if more than one conformation ID is listed, a corresponding model is a multiple-conformation docking model incorporating all the listed conformations. The cells in each table are colored according to its value from light for small values to dark for large. AUC values (positive docking model characteristic) are colored in green, while AUC standard errors (negative model characteristic) in red. Note, the AUC values shown here are just for illustration purposes. They were calculated on the whole set of 59 psVs and, therefore, are overoptimistic. The reliable AUC for the optimal model of 2219 estimated using the hold-out validation is reported in the Results section of the manuscript.
(PDF)

Figure S3 **Illustration of MDE performance for mAb 447-52D in the space of all single- and multiple-conformation docking models. (a)** Prediction AUC values for all tested docking models of mAb 447-52D calculated on the set of 59 psVs; **(b)** standard errors of prediction AUC values for corresponding docking models of mAb 447-52D. For both panels, ‘Start’ and ‘End’ are starting and ending positions of tested docking peptides; mAb conformation IDs correspond to the crystal structures in Table S1; if more than one conformation ID is listed, a corresponding model is a multiple-conformation docking model incorporating all the listed conformations. The cells in each table are colored according to its value from light for small values to dark for large. AUC values (positive docking model characteristic) are colored in green, while AUC standard errors (negative model characteristic) in red. Note, the AUC values shown here are just for illustration purposes. They were calculated on the whole set of 59 psVs and, therefore, are overoptimistic. The reliable AUC for the optimal model of 447-52D estimated using the hold-out validation is reported in the Results section of the manuscript.
(PDF)

Figure S4 **Patterns of masking effects in the V3 loop of gp120.** **(a)** availability of an epitope targeted by mAb 2219; **(b)** availability of an epitope targeted by mAb 447-52D; **(c)** availability of at least one of the two epitopes. In (a) and (b), green bars indicate strains predicted by MDE to possess a dynamic epitope of a given mAb, while red bars indicate strains with no such epitope. In (c), green bars indicate strains predicted to possess epitopes of at least one of the two mAbs, while the red bar indicates a strain, which does not have both epitopes. In (c), for each strain the lowest IC50 value of two mAbs is shown.
(PDF)

Figure S5 **Self- and cross-docking validation of the Flexible Peptide Docking protocol.** Root mean square deviation (RMSD, in Å) between FPD-predicted structures of the V3 peptides and their cognate crystallographic structures are shown for mAb 2219 (panels **a,** **c**) and 447-52D (panels **b,** **d**). RMSD values in panels **a** and **b** were calculated for backbone heavy atoms of the whole docked peptide. In contrast RMSD values in **c** and **d** were calculated only for backbone heavy atoms of the V3 regions covered by the predicted optimal docking peptides of each mAb (i.e. positions 10–13 for 2219, and 9–20 for 447-52D).
(PDF)

Figure S6 **Visualization of the V3 peptides MN (a), UG1033 (b), and UG29 (c) docked into the Fab of the mAb 2219 crystallized in complex with MN peptide (2B0S).** Structures derived experimentally by crystallography (green) and FPD-predicted structures (violet) are shown on the surface of the mAb 2219.
(PDF)

Table S1 **List of crystal structures of antibody-peptide complexes for mAbs 2219 and 447-52D used in the current study.**
(PDF)

Table S2 **Comparison of the Method of Dynamic Epitopes to the Signature Motif Method.**
(PDF)

Table S3 **Subtype specific estimates of epitope masking for mAb 2219 and 447-52D.**
(PDF)

Text S1 **Supplementary methods.**
(PDF)

Structure S1 **PDB-format structure of the V3 peptides MN (chains d, e, f, g, h, i, j, k, l, m), UG1033 (chains n, o, p, q, r, s, t, u, v, w), and UG29 (chains x, y, z, 1, 2, 3, 4, 5, 6, 7) predicted by docking into the Fab of the mAb 2219 (PDB 2B0S), as compared to the experimentally determined conformations of the same peptides extracted from PDB records 2B0S, 2B1A, and 2B1H (chains a, b, and c respectively).**
(PDB)
