# Supplementary figures and images for: Computational Prediction of Neutralization Epitopes Targeted by Human Anti-V3 HIV Monoclonal Antibodies
Source: PLoS One. 2014 Feb 25;9(2):e89987. doi: 10.1371/journal.pone.0089987 (PMC3934971; doi:10.1371/journal.pone.0089987)

Supplementary Figure S1

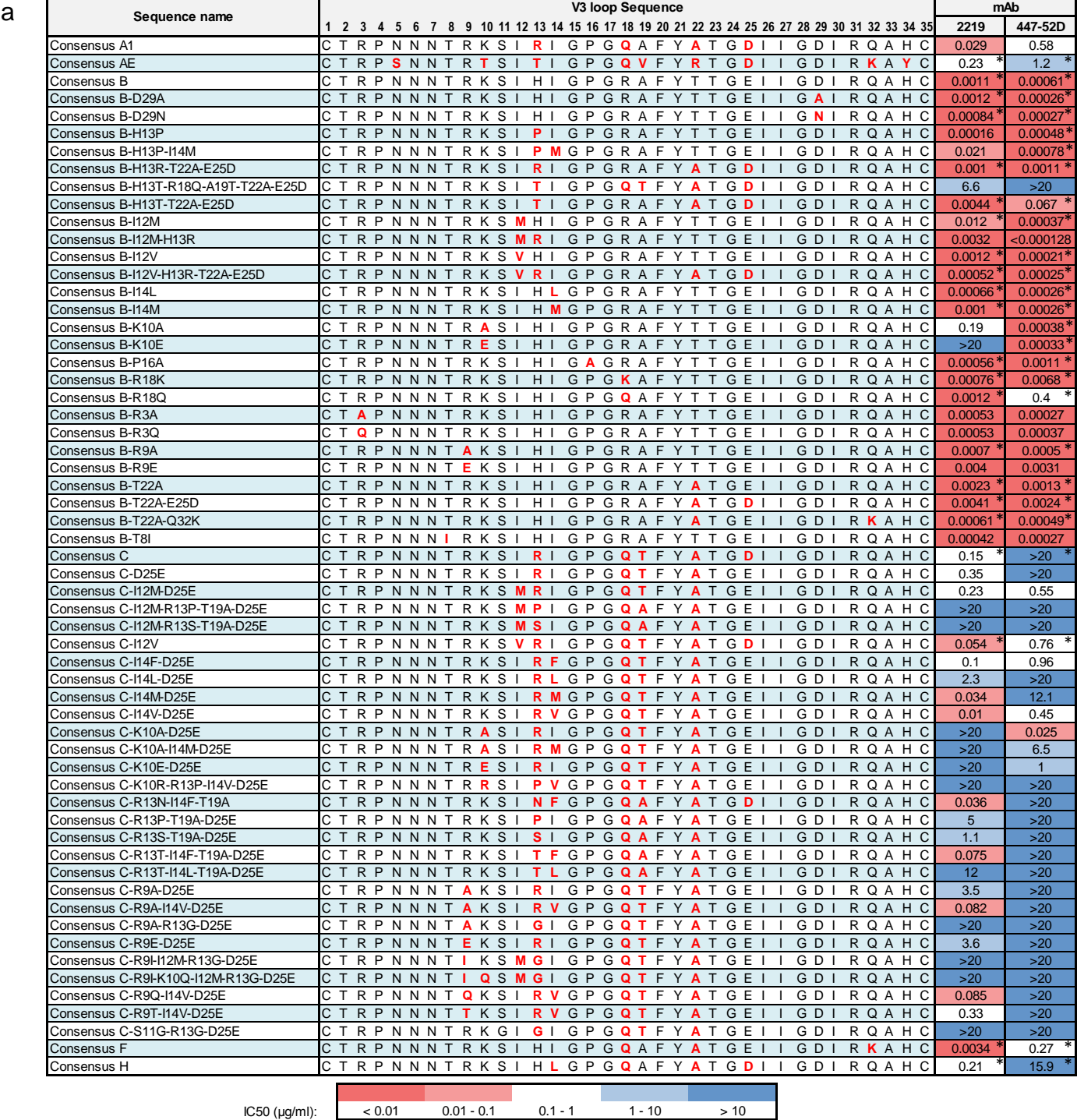

Supplement: Figure S1 — Neutralization of psVs containing various V3 loop sequences by mAbs 2219 and 447-52D. (a) Sequences of V3 loop and IC50 values (in µg/ml) of neutralization by each mAb are shown for 59 SF162 psVs. Amino acids of a V3 loop different from the consensus B sequence are shown in red. The V3 loop sequence is numbered according to the standard V3 numbering described elsewhere [14]. The IC50 value denoted as ‘>20’ represents a non-detectable level of neutralization at the range of concentrations used in the experiment. The IC50 data labeled with ‘*’ were derived from the previously published study [13]. The IC50 cells are colored according to its value from red to blue, where red background corresponds to high neutralization (small IC50 values) and blue to low neutralization (large IC50 values; see the color code legend in the bottom of the panel); (b) Normalized (to 100%) histogram of all IC50 values of neutralization from panel (a). The distribution has two distinct populations at concentrations <1 µg/ml and at the concentrations >20µg/ml. (PDF) [file pone.0089987.s001.pdf]

## Supplementary Figure S4

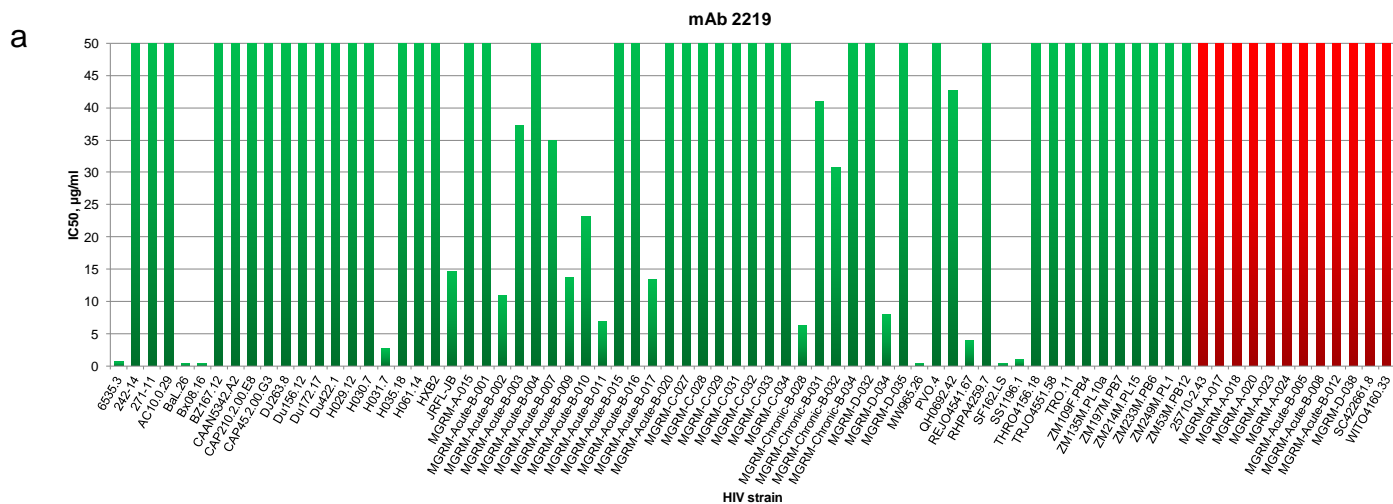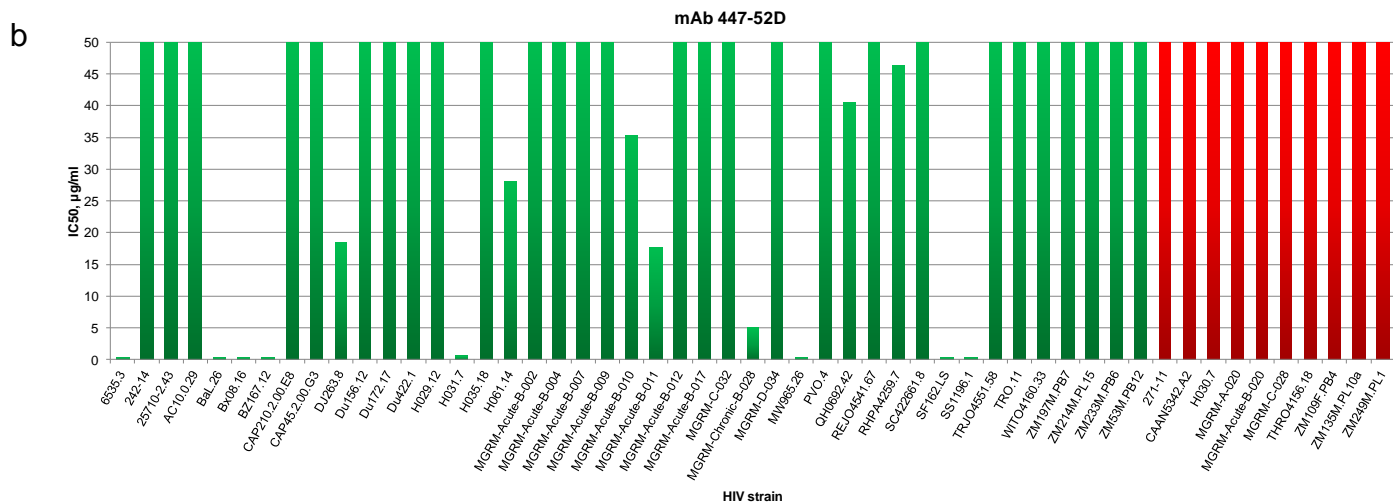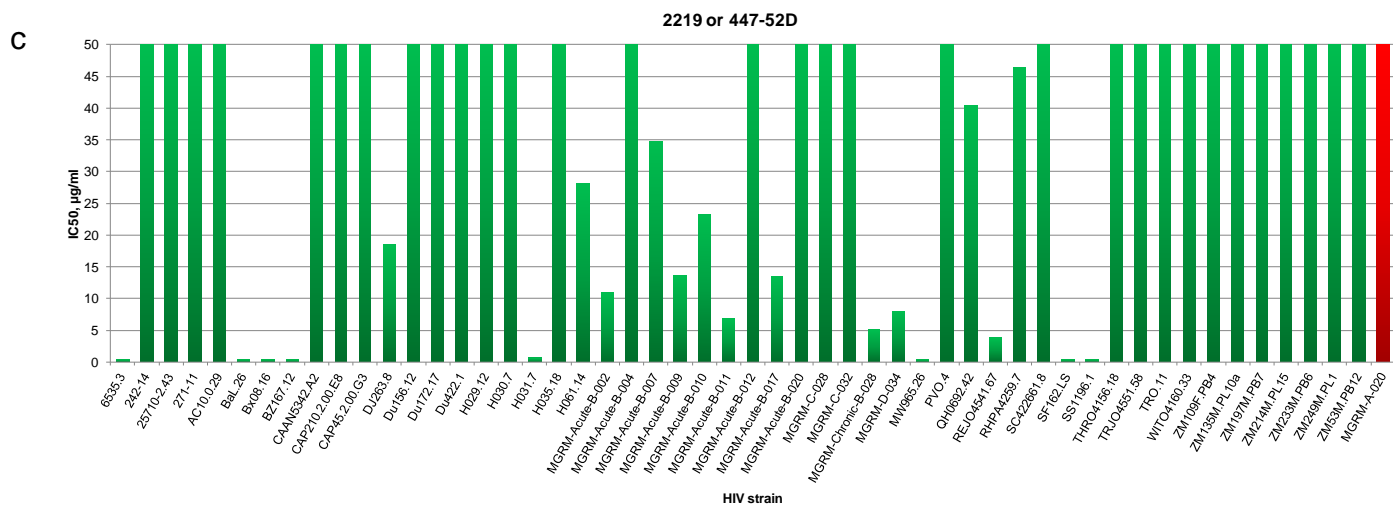

Supplement: Figure S4 — Patterns of masking effects in the V3 loop of gp120. (a) availability of an epitope targeted by mAb 2219; (b) availability of an epitope targeted by mAb 447-52D; (c) availability of at least one of the two epitopes. In (a) and (b), green bars indicate strains predicted by MDE to possess a dynamic epitope of a given mAb, while red bars indicate strains with no such epitope. In (c), green bars indicate strains predicted to possess epitopes of at least one of the two mAbs, while the red bar indicates a strain, which does not have both epitopes. In (c), for each strain the lowest IC50 value of two mAbs is shown. (PDF) [file pone.0089987.s004.pdf]

Supplementary Figure S6

a

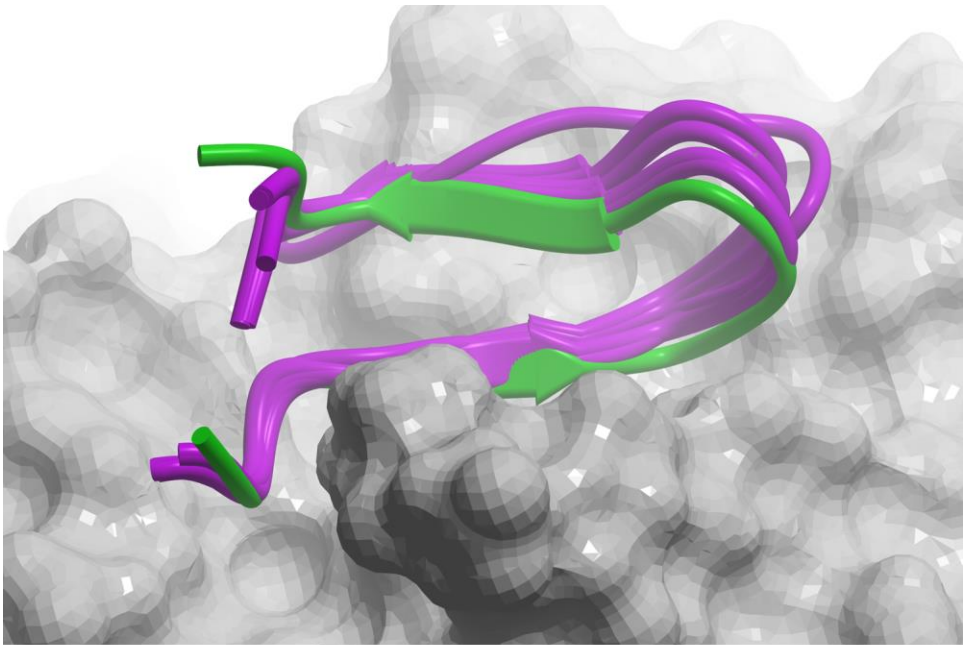

b

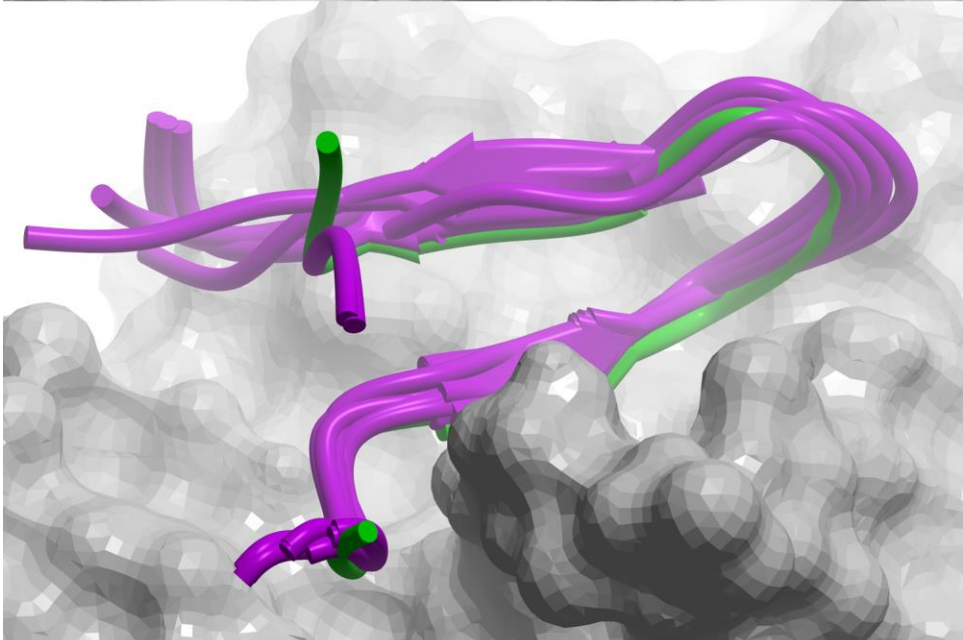

c

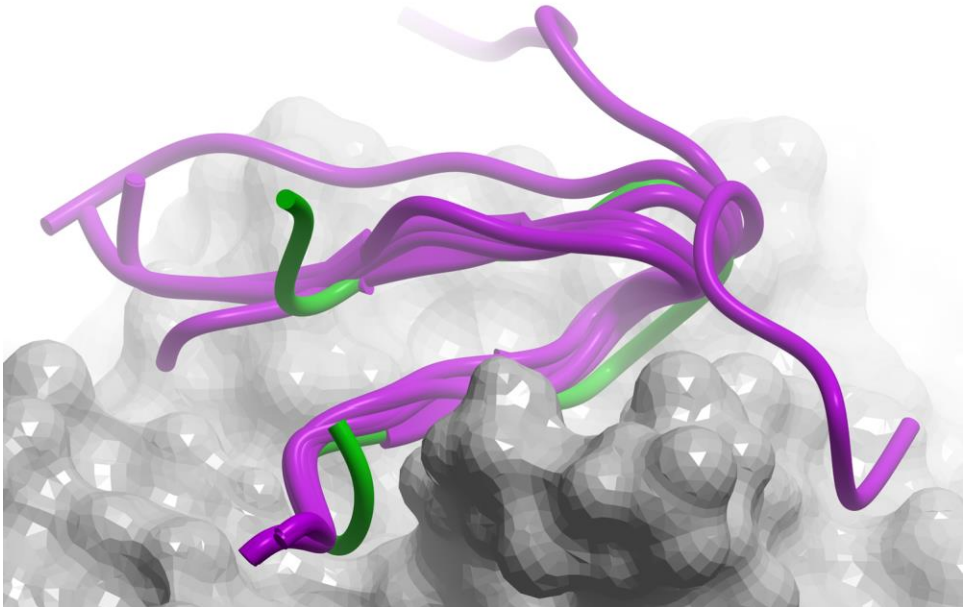

Supplement: Figure S6 — Visualization of the V3 peptides MN (a), UG1033 (b), and UG29 (c) docked into the Fab of the mAb 2219 crystallized in complex with MN peptide (2B0S). Structures derived experimentally by crystallography (green) and FPD-predicted structures (violet) are shown on the surface of the mAb 2219. (PDF) [file pone.0089987.s006.pdf]
