# Supplementary material for: Computational Prediction of Neutralization Epitopes Targeted by Human Anti-V3 HIV Monoclonal Antibodies
Source: PLoS One. 2014 Feb 25;9(2):e89987. doi: 10.1371/journal.pone.0089987 (PMC3934971; doi:10.1371/journal.pone.0089987)
Supplement: Figure S2 — Illustration of MDE performance for mAb 2219 in the space of all single- and multiple-conformation docking models. (a) prediction AUC values for all tested docking models of mAb 2219 calculated on the whole set of 59 psVs; (b) standard errors of prediction AUC values for corresponding tested docking models of mAb 2219. For both panels, ‘Start’ and ‘End’ are starting and ending positions of tested docking peptides; mAb conformation IDs correspond to the crystal structures in Table S1; if more than one conformation ID is listed, a corresponding model is a multiple-conformation docking model incorporating all the listed conformations. The cells in each table are colored according to its value from light for small values to dark for large. AUC values (positive docking model characteristic) are colored in green, while AUC standard errors (negative model characteristic) in red. Note, the AUC values shown here are just for illustration purposes. They were calculated on the whole set of 59 psVs and, therefore, are overoptimistic. The reliable AUC for the optimal model of 2219 estimated using the hold-out validation is reported in the Results section of the manuscript. (PDF) [file pone.0089987.s002.pdf]

Supplementary Figure S2

a

Docking peptide:		mAb 2219 conformation:						
Start	End	2B0S	2B1A	2B1H	2B0S 2B1A	2B1A 2B1H	2B0S 2B1H	2B0S 2B1A 2B1H
10	21	0.95	0.87	0.92	0.94	0.91	0.96	0.95
10	20	0.93	0.87	0.89	0.90	0.87	0.91	0.89
10	19	0.93	0.87	0.89	0.91	0.88	0.92	0.91
10	18	0.95	0.91	0.91	0.94	0.92	0.94	0.94
10	17	0.94	0.91	0.86	0.93	0.90	0.94	0.93
10	16	0.94	0.92	0.91	0.95	0.93	0.94	0.95
10	15	0.94	0.93	0.92	0.95	0.94	0.95	0.95
10	14	0.94	0.93	0.93	0.95	0.94	0.95	0.95
10	13	0.96	0.92	0.92	0.94	0.91	0.94	0.93
9	20	0.91	0.89	0.88	0.90	0.89	0.91	0.91
9	19	0.91	0.87	0.90	0.90	0.88	0.91	0.90
9	18	0.91	0.90	0.91	0.91	0.91	0.92	0.92
9	17	0.91	0.89	0.89	0.91	0.90	0.92	0.92
9	16	0.90	0.89	0.89	0.90	0.90	0.91	0.91
9	15	0.91	0.90	0.89	0.91	0.91	0.91	0.91
9	14	0.90	0.90	0.89	0.91	0.90	0.91	0.91
9	13	0.94	0.91	0.92	0.92	0.91	0.93	0.92
8	19	0.92	0.91	0.88	0.92	0.91	0.91	0.92
8	18	0.91	0.91	0.90	0.91	0.91	0.91	0.91
8	17	0.91	0.91	0.86	0.92	0.91	0.91	0.92
8	16	0.91	0.91	0.85	0.92	0.91	0.91	0.91
8	15	0.90	0.91	0.87	0.91	0.91	0.90	0.91
8	14	0.91	0.92	0.89	0.92	0.92	0.91	0.92
8	13	0.95	0.93	0.90	0.94	0.93	0.94	0.94
7	18	0.91	0.90	0.86	0.92	0.91	0.91	0.92
7	17	0.91	0.88	0.81	0.92	0.89	0.91	0.92
7	16	0.91	0.87	0.85	0.92	0.91	0.91	0.91
7	15	0.90	0.87	0.85	0.91	0.90	0.91	0.92
7	14	0.90	0.88	0.83	0.91	0.90	0.90	0.91
7	13	0.94	0.87	0.88	0.94	0.91	0.94	0.94
6	17	0.90	0.88	0.77	0.91	0.87	0.90	0.91
6	16	0.91	0.87	0.79	0.91	0.87	0.91	0.91
6	15	0.90	0.88	0.81	0.91	0.89	0.91	0.91
6	14	0.92	0.88	0.83	0.92	0.90	0.92	0.93
6	13	0.94	0.81	0.87	0.93	0.85	0.94	0.93
5	16	0.92	0.86	0.79	0.92	0.86	0.92	0.92
5	15	0.91	0.82	0.80	0.92	0.87	0.91	0.92
5	14	0.91	0.85	0.79	0.92	0.87	0.91	0.92
5	13	0.93	0.79	0.81	0.92	0.82	0.93	0.92
4	15	0.88	0.84	0.77	0.89	0.85	0.89	0.90
4	14	0.90	0.81	0.76	0.90	0.84	0.90	0.90
4	13	0.92	0.86	0.76	0.93	0.87	0.93	0.93
3	14	0.81	0.78	0.75	0.85	0.78	0.85	0.86
3	13	0.82	0.69	0.71	0.83	0.71	0.85	0.84
2	13	0.73	0.57	0.70	0.70	0.62	0.75	0.74

b

Docking peptide:		mAb 2219 conformation:						
Start	End	2B0S	2B1A	2B1H	2B0S 2B1A	2B1A 2B1H	2B0S 2B1H	2B0S 2B1A 2B1H
10	21	0.03	0.05	0.04	0.03	0.04	0.03	0.03
10	20	0.04	0.07	0.06	0.05	0.07	0.05	0.06
10	19	0.04	0.06	0.05	0.04	0.05	0.04	0.04
10	18	0.03	0.04	0.04	0.03	0.04	0.03	0.03
10	17	0.03	0.05	0.06	0.04	0.05	0.03	0.04
10	16	0.03	0.04	0.04	0.03	0.04	0.03	0.03
10	15	0.03	0.03	0.04	0.03	0.03	0.03	0.03
10	14	0.03	0.03	0.03	0.03	0.03	0.03	0.03
10	13	0.03	0.04	0.04	0.03	0.04	0.03	0.03
9	20	0.04	0.05	0.05	0.04	0.05	0.04	0.04
9	19	0.04	0.05	0.04	0.04	0.05	0.04	0.04
9	18	0.04	0.04	0.04	0.04	0.04	0.04	0.04
9	17	0.04	0.05	0.05	0.04	0.04	0.04	0.04
9	16	0.04	0.04	0.04	0.04	0.04	0.04	0.04
9	15	0.04	0.04	0.05	0.04	0.04	0.04	0.04
9	14	0.04	0.04	0.04	0.04	0.04	0.04	0.04
9	13	0.03	0.04	0.04	0.03	0.04	0.03	0.03
8	19	0.04	0.04	0.05	0.04	0.04	0.04	0.04
8	18	0.04	0.04	0.05	0.04	0.04	0.04	0.04
8	17	0.04	0.04	0.06	0.04	0.04	0.04	0.04
8	16	0.04	0.04	0.06	0.04	0.04	0.04	0.04
8	15	0.04	0.04	0.06	0.04	0.04	0.04	0.04
8	14	0.04	0.04	0.05	0.04	0.04	0.04	0.04
8	13	0.03	0.03	0.05	0.03	0.03	0.03	0.03
7	18	0.04	0.04	0.06	0.04	0.04	0.04	0.04
7	17	0.04	0.05	0.08	0.04	0.05	0.05	0.04
7	16	0.04	0.05	0.06	0.04	0.04	0.04	0.04
7	15	0.04	0.05	0.06	0.04	0.05	0.04	0.04
7	14	0.04	0.05	0.07	0.04	0.05	0.04	0.04
7	13	0.03	0.05	0.05	0.03	0.04	0.03	0.03
6	17	0.05	0.05	0.09	0.04	0.06	0.05	0.04
6	16	0.04	0.05	0.08	0.04	0.05	0.04	0.04
6	15	0.05	0.04	0.08	0.04	0.04	0.04	0.04
6	14	0.04	0.05	0.07	0.04	0.05	0.04	0.04
6	13	0.03	0.06	0.05	0.03	0.06	0.03	0.03
5	16	0.04	0.05	0.08	0.04	0.06	0.04	0.04
5	15	0.04	0.06	0.07	0.04	0.06	0.04	0.04
5	14	0.04	0.06	0.08	0.04	0.06	0.04	0.04
5	13	0.03	0.07	0.07	0.04	0.07	0.03	0.04
4	15	0.05	0.06	0.09	0.05	0.06	0.05	0.05
4	14	0.04	0.06	0.09	0.04	0.06	0.04	0.04
4	13	0.04	0.05	0.08	0.03	0.05	0.04	0.03
3	14	0.06	0.08	0.09	0.06	0.08	0.06	0.06
3	13	0.06	0.08	0.09	0.06	0.08	0.05	0.05
2	13	0.08	0.11	0.09	0.07	0.10	0.08	0.07
