# Supplementary material for: Computational Prediction of Neutralization Epitopes Targeted by Human Anti-V3 HIV Monoclonal Antibodies
Source: PLoS One. 2014 Feb 25;9(2):e89987. doi: 10.1371/journal.pone.0089987 (PMC3934971; doi:10.1371/journal.pone.0089987)
Supplement: Figure S3 — Illustration of MDE performance for mAb 447-52D in the space of all single- and multiple-conformation docking models. (a) Prediction AUC values for all tested docking models of mAb 447-52D calculated on the set of 59 psVs; (b) standard errors of prediction AUC values for corresponding docking models of mAb 447-52D. For both panels, ‘Start’ and ‘End’ are starting and ending positions of tested docking peptides; mAb conformation IDs correspond to the crystal structures in Table S1; if more than one conformation ID is listed, a corresponding model is a multiple-conformation docking model incorporating all the listed conformations. The cells in each table are colored according to its value from light for small values to dark for large. AUC values (positive docking model characteristic) are colored in green, while AUC standard errors (negative model characteristic) in red. Note, the AUC values shown here are just for illustration purposes. They were calculated on the whole set of 59 psVs and, therefore, are overoptimistic. The reliable AUC for the optimal model of 447-52D estimated using the hold-out validation is reported in the Results section of the manuscript. (PDF) [file pone.0089987.s003.pdf]

Supplementary Figure S3

a

Docking peptide:	Start	9	9	9	9	8	8	8	7	7	6
	End	22	21	20	19	21	20	19	20	19	19
mAb 447-52D conformation:	1Q1Jp	0.66	0.70	0.81	0.85	0.62	0.76	0.81	0.62	0.66	0.65
	1Q1Jq	0.72	0.70	0.88	0.84	0.62	0.72	0.76	0.59	0.64	0.62
	3C2Ap	0.82	0.85	0.81	0.85	0.82	0.81	0.82	0.69	0.76	0.69
	3C2Aq	0.81	0.83	0.83	0.83	0.62	0.60	0.68	0.53	0.57	0.68
	3GHBp	0.60	0.67	0.71	0.68	0.63	0.73	0.61	0.59	0.61	0.61
	3GHBq	0.71	0.76	0.68	0.69	0.69	0.60	0.72	0.59	0.65	0.63
	1Q1Jp 1Q1Jq	0.72	0.75	0.86	0.88	0.64	0.77	0.82	0.62	0.69	0.68
	1Q1Jp 3C2Ap	0.80	0.85	0.84	0.87	0.80	0.82	0.84	0.67	0.78	0.71
	1Q1Jp 3C2Aq	0.80	0.80	0.84	0.87	0.67	0.74	0.81	0.64	0.67	0.69
	1Q1Jp 3GHBp	0.67	0.71	0.82	0.83	0.66	0.78	0.77	0.63	0.65	0.69
	1Q1Jp 3GHBq	0.74	0.79	0.82	0.84	0.68	0.75	0.82	0.61	0.69	0.65
	1Q1Jq 3C2Ap	0.82	0.85	0.84	0.86	0.81	0.81	0.82	0.71	0.76	0.71
	1Q1Jq 3C2Aq	0.79	0.82	0.86	0.88	0.67	0.70	0.76	0.60	0.63	0.69
	1Q1Jq 3GHBp	0.68	0.71	0.83	0.81	0.66	0.75	0.70	0.61	0.64	0.67
	1Q1Jq 3GHBq	0.73	0.78	0.85	0.82	0.67	0.69	0.76	0.61	0.68	0.63
	3C2Ap 3C2Aq	0.81	0.86	0.83	0.86	0.81	0.79	0.81	0.68	0.74	0.71
	3C2Ap 3GHBp	0.77	0.82	0.81	0.85	0.80	0.81	0.79	0.68	0.71	0.69
	3C2Ap 3GHBq	0.79	0.85	0.81	0.85	0.80	0.80	0.81	0.68	0.76	0.69
	3C2Aq 3GHBp	0.73	0.78	0.81	0.78	0.68	0.70	0.65	0.61	0.61	0.68
	3C2Aq 3GHBq	0.77	0.82	0.81	0.82	0.71	0.64	0.74	0.60	0.63	0.68
	3GHBp 3GHBq	0.70	0.75	0.73	0.70	0.69	0.71	0.65	0.60	0.61	0.64
	1Q1Jp 1Q1Jq 3C2Ap	0.80	0.85	0.85	0.87	0.79	0.82	0.83	0.68	0.79	0.73
	1Q1Jp 1Q1Jq 3C2Aq	0.78	0.81	0.86	0.89	0.68	0.76	0.81	0.64	0.69	0.71
	1Q1Jp 1Q1Jq 3GHBp	0.70	0.74	0.85	0.87	0.68	0.79	0.78	0.63	0.67	0.72
	1Q1Jp 1Q1Jq 3GHBq	0.74	0.80	0.85	0.87	0.68	0.75	0.82	0.60	0.71	0.67
	1Q1Jp 3C2Ap 3C2Aq	0.80	0.85	0.85	0.87	0.79	0.82	0.83	0.68	0.77	0.73
	1Q1Jp 3C2Ap 3GHBp	0.77	0.82	0.84	0.86	0.78	0.83	0.82	0.68	0.73	0.71
	1Q1Jp 3C2Ap 3GHBq	0.78	0.85	0.84	0.87	0.79	0.82	0.84	0.67	0.78	0.70
	1Q1Jp 3C2Aq 3GHBp	0.74	0.77	0.84	0.84	0.68	0.77	0.77	0.64	0.66	0.72
	1Q1Jp 3C2Aq 3GHBq	0.78	0.82	0.84	0.86	0.70	0.75	0.81	0.63	0.68	0.69
	1Q1Jp 3GHBp 3GHBq	0.73	0.76	0.82	0.83	0.70	0.77	0.78	0.62	0.65	0.68
	1Q1Jq 3C2Ap 3C2Aq	0.81	0.85	0.85	0.87	0.80	0.81	0.81	0.70	0.74	0.74
	1Q1Jq 3C2Ap 3GHBp	0.78	0.83	0.83	0.86	0.79	0.81	0.79	0.69	0.71	0.72
	1Q1Jq 3C2Ap 3GHBq	0.79	0.85	0.83	0.86	0.79	0.81	0.81	0.70	0.76	0.71
	1Q1Jq 3C2Aq 3GHBp	0.74	0.78	0.84	0.84	0.69	0.73	0.71	0.63	0.63	0.71
	1Q1Jq 3C2Aq 3GHBq	0.77	0.82	0.84	0.86	0.69	0.69	0.77	0.63	0.66	0.69
	1Q1Jq 3GHBp 3GHBq	0.72	0.76	0.81	0.80	0.69	0.74	0.71	0.61	0.64	0.68
	3C2Ap 3C2Aq 3GHBp	0.78	0.83	0.83	0.85	0.80	0.80	0.79	0.68	0.70	0.72
	3C2Ap 3C2Aq 3GHBq	0.79	0.86	0.82	0.86	0.80	0.80	0.81	0.69	0.74	0.71
	3C2Ap 3GHBp 3GHBq	0.77	0.84	0.81	0.85	0.78	0.81	0.79	0.68	0.71	0.68
	3C2Aq 3GHBp 3GHBq	0.74	0.80	0.80	0.78	0.71	0.70	0.68	0.62	0.60	0.69
	1Q1Jp 1Q1Jq 3C2Ap 3C2Aq	0.80	0.85	0.86	0.88	0.78	0.82	0.83	0.68	0.77	0.75
	1Q1Jp 1Q1Jq 3C2Ap 3GHBp	0.78	0.83	0.84	0.87	0.78	0.83	0.82	0.68	0.74	0.74
	1Q1Jp 1Q1Jq 3C2Ap 3GHBq	0.79	0.85	0.85	0.87	0.78	0.82	0.83	0.67	0.78	0.72
	1Q1Jp 1Q1Jq 3C2Aq 3GHBp	0.75	0.78	0.86	0.87	0.70	0.78	0.79	0.64	0.67	0.75
	1Q1Jp 1Q1Jq 3C2Aq 3GHBq	0.78	0.82	0.86	0.88	0.70	0.75	0.81	0.63	0.70	0.71
	1Q1Jp 1Q1Jq 3GHBp 3GHBq	0.74	0.77	0.84	0.86	0.70	0.79	0.79	0.62	0.67	0.71
	1Q1Jp 3C2Ap 3C2Aq 3GHBp	0.78	0.83	0.85	0.87	0.78	0.83	0.81	0.68	0.73	0.73
	1Q1Jp 3C2Ap 3C2Aq 3GHBq	0.78	0.85	0.84	0.87	0.79	0.82	0.83	0.68	0.77	0.72
	1Q1Jp 3C2Ap 3GHBp 3GHBq	0.77	0.83	0.84	0.87	0.78	0.83	0.82	0.67	0.73	0.70
	1Q1Jp 3C2Aq 3GHBp 3GHBq	0.75	0.80	0.83	0.84	0.71	0.77	0.78	0.64	0.65	0.72
	1Q1Jq 3C2Ap 3C2Aq 3GHBp	0.79	0.84	0.84	0.86	0.79	0.81	0.79	0.69	0.71	0.74
	1Q1Jq 3C2Ap 3C2Aq 3GHBq	0.80	0.86	0.84	0.86	0.79	0.81	0.81	0.70	0.75	0.73
	1Q1Jq 3C2Ap 3GHBp 3GHBq	0.78	0.84	0.82	0.85	0.78	0.81	0.79	0.69	0.72	0.71
	1Q1Jq 3C2Aq 3GHBp 3GHBq	0.75	0.80	0.83	0.84	0.70	0.74	0.72	0.63	0.63	0.72
	3C2Ap 3C2Aq 3GHBp 3GHBq	0.77	0.84	0.82	0.85	0.78	0.80	0.79	0.69	0.70	0.71
	1Q1Jp 1Q1Jq 3C2Ap 3C2Aq 3GHBp	0.79	0.83	0.85	0.87	0.77	0.83	0.81	0.69	0.73	0.76
	1Q1Jp 1Q1Jq 3C2Ap 3C2Aq 3GHBq	0.79	0.85	0.85	0.88	0.78	0.82	0.83	0.68	0.77	0.74
	1Q1Jp 1Q1Jq 3C2Ap 3GHBp 3GHBq	0.78	0.84	0.84	0.87	0.77	0.83	0.82	0.68	0.74	0.73
	1Q1Jp 1Q1Jq 3C2Aq 3GHBp 3GHBq	0.76	0.80	0.85	0.87	0.71	0.78	0.79	0.64	0.67	0.74
	1Q1Jp 3C2Ap 3C2Aq 3GHBp 3GHBq	0.78	0.84	0.84	0.87	0.78	0.83	0.81	0.69	0.73	0.73
	1Q1Jq 3C2Ap 3C2Aq 3GHBp 3GHBq	0.79	0.85	0.83	0.86	0.78	0.81	0.79	0.70	0.71	0.74
	1Q1Jp 1Q1Jq 3C2Ap 3C2Aq 3GHBp	0.79	0.84	0.85	0.87	0.77	0.83	0.81	0.69	0.73	0.75

b

Docking peptide:	Start	9	9	9	9	8	8	8	7	7	6
	End	22	21	20	19	21	20	19	20	19	19
mAb 447-52D conformation:	1Q1Jp	0.07	0.07	0.06	0.05	0.08	0.06	0.06	0.08	0.07	0.07
	1Q1Jq	0.07	0.07	0.05	0.05	0.08	0.07	0.07	0.08	0.08	0.08
	3C2Ap	0.06	0.05	0.06	0.05	0.06	0.06	0.06	0.07	0.06	0.07
	3C2Aq	0.06	0.05	0.06	0.06	0.08	0.08	0.07	0.08	0.08	0.07
	3GHBp	0.08	0.08	0.07	0.07	0.07	0.07	0.08	0.08	0.08	0.08
	3GHBq	0.07	0.07	0.07	0.08	0.07	0.08	0.07	0.08	0.08	0.08
	1Q1Jp 1Q1Jq	0.07	0.07	0.05	0.04	0.08	0.06	0.06	0.08	0.07	0.07
	1Q1Jp 3C2Ap	0.07	0.05	0.06	0.05	0.07	0.06	0.06	0.08	0.06	0.07
	1Q1Jp 3C2Aq	0.06	0.06	0.05	0.05	0.08	0.07	0.06	0.08	0.07	0.07
	1Q1Jp 3GHBp	0.08	0.07	0.06	0.05	0.07	0.06	0.06	0.08	0.07	0.07
	1Q1Jp 3GHBq	0.07	0.06	0.06	0.05	0.08	0.07	0.06	0.08	0.07	0.08
	1Q1Jq 3C2Ap	0.06	0.05	0.06	0.05	0.06	0.06	0.06	0.07	0.07	0.07
	1Q1Jq 3C2Aq	0.06	0.06	0.05	0.05	0.08	0.07	0.07	0.08	0.08	0.07
	1Q1Jq 3GHBp	0.07	0.07	0.06	0.06	0.07	0.07	0.07	0.08	0.08	0.07
	1Q1Jq 3GHBq	0.07	0.06	0.05	0.06	0.08	0.08	0.07	0.08	0.07	0.08
	3C2Ap 3C2Aq	0.07	0.05	0.06	0.05	0.06	0.06	0.06	0.07	0.07	0.07
	3C2Ap 3GHBp	0.07	0.06	0.06	0.06	0.06	0.06	0.06	0.07	0.07	0.07
	3C2Ap 3GHBq	0.07	0.05	0.06	0.05	0.06	0.06	0.06	0.07	0.07	0.07
	3C2Aq 3GHBp	0.07	0.06	0.06	0.06	0.07	0.07	0.08	0.08	0.08	0.07
	3C2Aq 3GHBq	0.07	0.06	0.06	0.06	0.07	0.08	0.07	0.08	0.08	0.07
	3GHBp 3GHBq	0.07	0.07	0.07	0.08	0.07	0.07	0.08	0.08	0.08	0.08
	1Q1Jp 1Q1Jq 3C2Ap	0.06	0.05	0.06	0.05	0.07	0.06	0.06	0.08	0.06	0.07
	1Q1Jp 1Q1Jq 3C2Aq	0.07	0.06	0.05	0.04	0.08	0.06	0.06	0.08	0.07	0.07
	1Q1Jp 1Q1Jq 3GHBp	0.07	0.07	0.05	0.05	0.07	0.06	0.06	0.08	0.07	0.07
	1Q1Jp 1Q1Jq 3GHBq	0.07	0.06	0.05	0.05	0.08	0.06	0.06	0.08	0.07	0.07
	1Q1Jp 3C2Ap 3C2Aq	0.06	0.05	0.06	0.05	0.07	0.06	0.06	0.08	0.07	0.07
	1Q1Jp 3C2Ap 3GHBp	0.07	0.06	0.06	0.05	0.07	0.06	0.06	0.07	0.07	0.07
	1Q1Jp 3C2Ap 3GHBq	0.07	0.05	0.06	0.05	0.07	0.06	0.06	0.08	0.06	0.07
	1Q1Jp 3C2Aq 3GHBp	0.07	0.06	0.05	0.05	0.07	0.06	0.06	0.08	0.07	0.07
	1Q1Jp 3C2Aq 3GHBq	0.07	0.06	0.05	0.05	0.07	0.07	0.06	0.08	0.07	0.07
	1Q1Jp 3GHBp 3GHBq	0.07	0.07	0.06	0.06	0.07	0.06	0.06	0.08	0.07	0.07
	1Q1Jq 3C2Ap 3C2Aq	0.06	0.05	0.06	0.05	0.06	0.06	0.06	0.07	0.07	0.07
	1Q1Jq 3C2Ap 3GHBp	0.07	0.06	0.06	0.05	0.06	0.06	0.06	0.07	0.07	0.07
	1Q1Jq 3C2Ap 3GHBq	0.06	0.05	0.06	0.05	0.06	0.06	0.06	0.07	0.07	0.07
	1Q1Jq 3C2Aq 3GHBp	0.07	0.06	0.05	0.06	0.07	0.07	0.07	0.08	0.08	0.07
	1Q1Jq 3C2Aq 3GHBq	0.07	0.06	0.05	0.05	0.08	0.07	0.06	0.08	0.08	0.07
	1Q1Jq 3GHBp 3GHBq	0.07	0.07	0.06	0.06	0.07	0.07	0.07	0.08	0.08	0.07
	3C2Ap 3C2Aq 3GHBp	0.07	0.05	0.06	0.05	0.06	0.06	0.06	0.07	0.07	0.07
	3C2Ap 3C2Aq 3GHBq	0.07	0.05	0.06	0.05	0.06	0.06	0.06	0.07	0.07	0.07
	3C2Ap 3GHBp 3GHBq	0.07	0.05	0.06	0.06	0.07	0.06	0.06	0.07	0.07	0.07
	3C2Aq 3GHBp 3GHBq	0.07	0.06	0.06	0.06	0.07	0.07	0.08	0.08	0.08	0.07
	1Q1Jp 1Q1Jq 3C2Ap 3C2Aq	0.06	0.05	0.05	0.05	0.07	0.06	0.06	0.08	0.07	0.07
	1Q1Jp 1Q1Jq 3C2Ap 3GHBp	0.07	0.05	0.06	0.05	0.07	0.06	0.06	0.07	0.07	0.07
	1Q1Jp 1Q1Jq 3C2Ap 3GHBq	0.06	0.05	0.06	0.05	0.07	0.06	0.06	0.08	0.06	0.07
	1Q1Jp 1Q1Jq 3C2Aq 3GHBp	0.07	0.06	0.05	0.05	0.07	0.06	0.06	0.08	0.07	0.07
	1Q1Jp 1Q1Jq 3C2Aq 3GHBq	0.07	0.06	0.05	0.05	0.07	0.07	0.06	0.08	0.07	0.07
	1Q1Jp 1Q1Jq 3GHBp 3GHBq	0.07	0.07	0.06	0.05	0.07	0.06	0.06	0.08	0.07	0.07
	1Q1Jp 3C2Ap 3C2Aq 3GHBp	0.07	0.05	0.06	0.05	0.07	0.06	0.06	0.07	0.07	0.07
	1Q1Jp 3C2Ap 3C2Aq 3GHBq	0.07	0.05	0.06	0.05	0.07	0.06	0.06	0.08	0.07	0.07
	1Q1Jp 3C2Ap 3GHBp 3GHBq	0.07	0.05	0.06	0.05	0.07	0.06	0.06	0.08	0.07	0.07
	1Q1Jp 3C2Aq 3GHBp 3GHBq	0.07	0.06	0.05	0.05	0.07	0.07	0.06	0.08	0.08	0.07
	1Q1Jq 3C2Ap 3C2Aq 3GHBp	0.07	0.05	0.06	0.05	0.06	0.06	0.06	0.07	0.07	0.07
	1Q1Jq 3C2Ap 3C2Aq 3GHBq	0.06	0.05	0.06	0.05	0.06	0.06	0.06	0.07	0.07	0.07
	1Q1Jq 3C2Ap 3GHBp 3GHBq	0.07	0.05	0.06	0.05	0.07	0.06	0.06	0.07	0.07	0.07
	1Q1Jq 3C2Aq 3GHBp 3GHBq	0.07	0.06	0.06	0.06	0.07	0.07	0.07	0.08	0.08	0.07
	3C2Ap 3C2Aq 3GHBp 3GHBq	0.07	0.05	0.06	0.05	0.07	0.06	0.06	0.07	0.07	0.07
	1Q1Jp 1Q1Jq 3C2Ap 3C2Aq 3GHBp	0.07	0.05	0.05	0.05	0.07	0.06	0.06	0.07	0.07	0.07
	1Q1Jp 1Q1Jq 3C2Ap 3C2Aq 3GHBq	0.06	0.05	0.06	0.05	0.07	0.06	0.06	0.08	0.07	0.07
	1Q1Jp 1Q1Jq 3C2Ap 3GHBp 3GHBq	0.07	0.05	0.06	0.05	0.07	0.06	0.06	0.08	0.07	0.07
	1Q1Jp 1Q1Jq 3C2Aq 3GHBp 3GHBq	0.07	0.06	0.05	0.05	0.07	0.06	0.06	0.08	0.07	0.07
	1Q1Jp 3C2Ap 3C2Aq 3GHBp 3GHBq	0.07	0.05	0.06	0.05	0.07	0.06	0.06	0.07	0.07	0.07
	1Q1Jq 3C2Ap 3C2Aq 3GHBp 3GHBq	0.06	0.05	0.06	0.05	0.07	0.06	0.06	0.07	0.07	0.07
	1Q1Jp 1Q1Jq 3C2Ap 3C2Aq 3GHBp	0.06	0.05	0.06	0.05	0.07	0.06	0.06	0.07	0.07	0.07
