# Supplementary material for: Computational Prediction of Neutralization Epitopes Targeted by Human Anti-V3 HIV Monoclonal Antibodies
Source: PLoS One. 2014 Feb 25;9(2):e89987. doi: 10.1371/journal.pone.0089987 (PMC3934971; doi:10.1371/journal.pone.0089987)
Supplement: Figure S5 — Self- and cross-docking validation of the Flexible Peptide Docking protocol. Root mean square deviation (RMSD, in Å) between FPD-predicted structures of the V3 peptides and their cognate crystallographic structures are shown for mAb 2219 (panels a, c) and 447-52D (panels b, d). RMSD values in panels a and b were calculated for backbone heavy atoms of the whole docked peptide. In contrast RMSD values in c and d were calculated only for backbone heavy atoms of the V3 regions covered by the predicted optimal docking peptides of each mAb (i.e. positions 10–13 for 2219, and 9–20 for 447-52D). (PDF) [file pone.0089987.s005.pdf]

Supplementary Figure S5

a

	MN	UG1033	UR29	Self-docking	Cross-docking
**2B0S**	1.41	3.04	5.41	1.41	4.23
**2B1A**	1.57	3.09	4.79	3.09	3.18
**2B1H**	1.65	3.72	4.74	4.74	2.69
			**Average:**	3.08	3.37

b

	MN	UG1033	W2RW020 (10aa)	W2RW020 (9aa)	Self-docking	Cross-docking
**1Q1Jp**	2.08	5.70	1.48	1.66	2.08	2.95
**1Q1Jq**	1.78	7.07	3.20	1.68	1.78	3.98
**3C2Ap**	1.69	4.65	3.04	1.19	4.65	1.98
**3C2Aq**	1.79	2.70	2.22	1.65	2.70	1.89
**3GHBp**	3.01	6.11	1.76	1.55	1.76	3.56
**3GHBq**	2.52	6.75	2.93	2.31	2.31	4.06
				**Average:**	2.55	3.07

c

	MN	UG1033	UR29	Self-docking	Cross-docking
**2B0S**	0.41	0.65	0.72	0.41	0.68
**2B1A**	0.49	0.55	0.65	0.55	0.57
**2B1H**	0.60	0.65	0.55	0.55	0.63
			**Average:**	0.50	0.63

d

	MN	UG1033	W2RW020 (10aa)	W2RW020 (9aa)	Self-docking	Cross-docking
**1Q1Jp**	2.08	3.52	1.48	1.66	2.08	2.22
**1Q1Jq**	1.78	5.70	3.20	1.68	1.78	3.53
**3C2Ap**	1.69	1.89	3.04	1.19	1.89	1.98
**3C2Aq**	1.79	1.35	2.22	1.65	1.35	1.89
**3GHBp**	3.01	4.20	1.76	1.55	1.76	2.92
**3GHBq**	2.52	2.92	2.93	2.31	2.31	2.79
				**Average:**	1.86	2.55
