# Supplementary material for: Computational Prediction of Neutralization Epitopes Targeted by Human Anti-V3 HIV Monoclonal Antibodies
Source: PLoS One. 2014 Feb 25;9(2):e89987. doi: 10.1371/journal.pone.0089987 (PMC3934971; doi:10.1371/journal.pone.0089987)
Supplement: Table S1 — List of crystal structures of antibody-peptide complexes for mAbs 2219 and 447-52D used in the current study. (PDF) [file pone.0089987.s007.pdf]

Supplementary Table S1: List of crystal structures of antibody-peptide complexes for mAbs 2219 and 447-52D used in the current study.

mAb	Crystal Structure PDB ID	Viral Strain of gp120 Peptide	Sequence of gp120 peptide	Reference	Conformation ID
2219	2B0S	MN	KRKRIHIGPGRAFYTT	Stanfield et al. 2006	2B0S
2219	2B1A	UG1033	TRKSIHLGPGRAFYAT	Stanfield et al. 2006	2B1A
2219	2B1H	UR29	TKKSIKIRPRQAFYAT	Stanfield et al. 2006	2B1H
447-52D	3C2A	UG1033	KSIHLGPGRAFYA	Dhillon et al. 2008	3C2Ap
447-52D	3C2A	UG1033	KSIHLGPGRAFYA	Dhillon et al. 2008	3C2Aq
447-52D	1Q1J	MN	KRIHIGPGRA	Stanfield et al. 2004	1Q1Jp
447-52D	1Q1J	MN	KRIHIGPGRA	Stanfield et al. 2004	1Q1Jq
447-52D	3GHB	W2RW020	KGVRIGPGQA	Burke et al. 2009	3GHBp
447-52D	3GHB	W2RW020	KGVRIGPGQ	Burke et al. 2009	3GHBq

Note: Crystal Structure PDB ID is a Protein Data Bank (http://www.rcsb.org) identification code of an antibody-peptide complex. Conformation ID is the antibody conformation identification code used in the text and figures of this paper.
