# Supplementary material for: Computational Prediction of Neutralization Epitopes Targeted by Human Anti-V3 HIV Monoclonal Antibodies
Source: PLoS One. 2014 Feb 25;9(2):e89987. doi: 10.1371/journal.pone.0089987 (PMC3934971; doi:10.1371/journal.pone.0089987)
Supplement: Table S2 — Comparison of the Method of Dynamic Epitopes to the Signature Motif Method. (PDF) [file pone.0089987.s008.pdf]

Supplementary Table S2: Comparison of the Method of Dynamic Epitopes to the Signature Motif Method.

**Method**	**Signature Motif Method (SMM)**			**Method of Dynamic Epitopes (MDE)**		
**mAb**	**2219**	**447-52D**	**Average**	**2219**	**447-52D**	**Average**
Accuracy (ACC)	0.78	0.75	0.76	0.93	0.82	0.87
Matthews Correlation Coefficient (MCC)	0.53	0.59	0.56	0.82	0.63	0.72
