# Supplementary material for: Computational Prediction of Neutralization Epitopes Targeted by Human Anti-V3 HIV Monoclonal Antibodies
Source: PLoS One. 2014 Feb 25;9(2):e89987. doi: 10.1371/journal.pone.0089987 (PMC3934971; doi:10.1371/journal.pone.0089987)
Supplement: Table S3 — Subtype specific estimates of epitope masking for mAb 2219 and 447-52D. (PDF) [file pone.0089987.s009.pdf]

Supplementary Table S3: Subtype specific estimates of epitope masking for mAb 2219 and 447-52D.

(a) Subtype specific masking of 2219 epitope

Subtype	A	B	C	D	G	CRF01_AE	CRF02_AG	Total
Number of viruses tested	7	43	21	4	0	0	2	77
Number of viruses with epitope	2	38	20	3	0	0	2	65
Number of viruses with masked epitope	2	19	19	2	0	0	2	44
Masked epitopes percentage	100.0	50.0	95.0	66.7	n/a	n/a	100.0	67.7

(b) Subtype specific masking of 447-52D epitope

Subtype	A	B	C	D	G	CRF01_AE	CRF02_AG	Total
Number of viruses tested	2	32	16	1	0	0	2	53
Number of viruses with epitope	1	28	12	1	0	0	1	43
Number of viruses with masked epitope	0	15	11	1	0	0	1	28
Masked epitopes percentage	0.0	53.6	91.7	100.0	n/a	n/a	100.0	65.1

(c) Subtype specific masking of both 2219 and 447-52D epitopes

Subtype	A	B	C	D	G	CRF01_AE	CRF02_AG	Total
Number of viruses tested	2	32	16	1	0	0	2	53
Number of viruses with at least one epitope	1	32	16	1	0	0	2	52
Number of viruses with masked epitopes	0	14	15	0	0	0	2	31
Masked epitopes percentage	0.0	43.8	93.8	0.0	n/a	n/a	100.0	59.6

Note: Panel (c) shows subtype specific masking of both epitopes: i.e. value 43.8% for subtype B suggests that in 56.2 (=100 - 43.8) percent of cases at least one of the two epitopes is unmasked. Bear in mind that the sample size for the subtypes A, D, G, and CRF01_AE and CRF02_AG is very small, and therefore, no conclusion should be made based on the their specific numbers.
