# Supplementary material for: Computational Prediction of Neutralization Epitopes Targeted by Human Anti-V3 HIV Monoclonal Antibodies
Source: PLoS One. 2014 Feb 25;9(2):e89987. doi: 10.1371/journal.pone.0089987 (PMC3934971; doi:10.1371/journal.pone.0089987)
Supplement: Text S1 — Supplementary methods. (PDF) [file pone.0089987.s010.pdf]

SUPPLEMENTARY METHODS

1. Selection of the threshold IC_{50} of psVs' neutralization

In order to select an optimal threshold IC_{50} value discriminating between those psVs that are neutralized by a given mAb *in vitro* and those that are not, we studied a distribution of all the experimentally measured IC_{50} values across the tested psVs (Supplementary Fig. S1). We found that this distribution has two distinct populations: one at concentrations below 1 µg/ml and another, at concentrations >20µg/ml. Only few values are recorded between 1 and 20µg/ml. This finding suggests that the reasonable threshold IC_{50} could be any number between 1 and 20µg/ml without significant differences in the results. Accordingly, in this study we selected a threshold IC_{50} value of 20µg/ml.

2. Evaluation of docking models based on single and multiple mAb conformations

While the V3 loop has a conserved ß-hairpin fold, it can adopt many different ß-hairpin conformations [1] and even more conformations in which side-chains differ [2,3,4,5]. This domain is therefore considerably flexible. Consequently, multiple ligand-bound conformations of the same mAb crystallized in complex with different V3 peptides vary in their structure. These variations may be significant for assessing the recognition of a specific HIV-1 strain (or strains) by a mAb. In other words, a given mAb conformation could be optimal for prediction of the presence of the neutralization epitope in one strain, while being not optimal for detecting it in another. Usage of all available mAb conformations (when the *docking peptide* in question is docked to each conformation separately and the minimal binding energy is selected) in MDE can potentially solve this problem. However, it would substantially increase the computational expense of the method. To address all these issues, in addition to considering each individual mAb conformation (see Methods), we performed an analysis of each possible combination of conformations according to the following strategy.

First, for a given mAb, all models based on individual mAb conformations were tested and the best single-conformation model was selected (see Methods). Second, all models based on multiple conformations were tested and the best multiple-conformation docking model was selected. Finally, the AUC of the best multiple-conformation model was statistically compared to the AUC of the best single-conformation model. If the multiple-conformation model had failed to significantly improve the quality of prediction comparing to the single-conformation model, the simpler single-conformation model was selected as optimal. Otherwise, the optimal model was the model with higher performance.

In the current study, usage of multiple-conformation models failed to significantly improve the performance of MDE for either of the two mAbs and therefore, the single-conformation models were selected as optimal (see Results). Nevertheless, the description of the analysis assessing both single- and multiple-conformation models is presented here for generality, as it could be crucial for optimization of MDE for other Abs.

3. Comparison of the performance of MDE and SMM

In order to compare the performance of MDE to the previously published SMM we used the same set of 59 psVs. We applied the following two comparison criteria: a) accuracy, also known as the proportion of correct classifications, defined as $ACC = \frac{(TP+TN)}{(TP+TN+FP+FN)}$; and b) Matthews correlation coefficient MCC, which is a more balanced measure of the quality of a binary classification (comparing to the accuracy) [6] and is defined as $MCC = \frac{(TP*TN-FP*FN)}{\sqrt{(TP+FP)*(TP+FN)*(TN+FP)*(TN+FN)}}$, where TP is the number of true positives, TN is the number of true negatives, FP is the number of false positives, and FN is the number of false negatives. The comparison is summarized on the Supplementary Table S2. These data demonstrate that the Method of Dynamic Epitopes substantially surpasses the Signature Motif Method in its quality of prediction.

4. Estimates of epitope conservation for the mAbs 2219 and 447-52D

Sequences of all strains of the five subtypes (A, B, C, D, G) and two CRFs (CRF01_AE and CRF02_AG) were extracted from the Los Alamos National Laboratory HIV Database. Problematic sequences (e.g., sequences with non-amino-acid symbols) were filtered out. Only one sequence per patient was randomly selected to avoid bias toward specific patients with large number of sequences. Presence of neutralization epitopes of either of the two mAbs in each strain in the resulting dataset was then predicted using MDE as following:

1. Two docking peptides were assigned for each sequence: one for 2219 and one for 447-52D. Starting and ending positions of each docking peptide on the V3 sequence of a query strain were selected according to the optimal docking models predefined for each of these mAbs (i.e., positions 10 and 13 for 2219, and 9 and 20 for 447-52D).
2. Presence of neutralization epitopes in strains with corresponding docking peptide sequences identical to the sequences of 59 psVs was set based on the experimentally determined neutralization for these psVs. For example, say a given strain X has a V3 loop sequence ^{1}CTRPNNNTRKSIHIGPGRAFYATGDIVGDIRQAHC^{35}, the 2219 docking peptide for this sequence is ^{10}KSIH^{13}. According to the set of 59 psVs, if a strain has ^{10}KSIH^{13} pattern in the V3 loop, it is neutralized detectably by 2219 in the absence of the masking effects (Supplementary Fig. S1). Therefore, the strain X is right away predicted to contain the 2219 mAb dynamic epitope. This step was performed in order to improve the accuracy of the estimates.
3. All the strains, which docking peptide sequences differ from those with the known neutralization, were docked into the mAbs and free energies of binding were calculated (according to the MDE protocol).
4. A threshold for the free energy of binding, used to discriminate strains that possess a dynamic neutralization epitope of a given mAb from the rest of the set, was selected based on the closest-to-(0,1) criterion [7] on the training set of 59 psVs (see Supplementary Figure S7 below). Specifically, the

following threshold energies were selected: E_{2219} = -31.469, 95% CI_{2219} (-32.920; -30.017), and $E_{447-52D}$ = -62.790, 95% $CI_{447-52D}$ (-65.928; -59.652), all in kcal/mol according to the ICM scoring function.

From that point onward, the estimates of epitope conservation were determined exactly as described in our previous studies [8,9], modified with the most recent WHO data on HIV-1 global prevalence [10]. The size of the sets of the assessed sequences for each of the subtypes and CRFs was the following (numbers are shown for mAb 2219/ mAb 447): 1883/1854 for subtype A, 12396/11636 for subtype B, 4282/4263 for subtype C, 2130/2117 for subtype D, 587/578 for subtype G, 2476/2440 for CRF01_AE, and 1132/1123 for CRF02_AG. Note, that the numbers of sequences are slightly different between the sets used for each of the two mAbs as sequences with insertions/deletions in the region of V3 covered by the corresponding docking peptides of each mAb were excluded from the analysis. The reason for this additional filtering is that the selection of a precise continuous epitope region on a sequence with insertions/deletions in that region of V3 is challenging from the structural point of view. However, due to the fact that insertions/deletions occur very rarely in V3, the effect of the exclusion of these sequences should be negligible.

5. Estimates of epitope masking effects and effective breadths of reactivity of the mAbs 2219 and 447-52D

The analysis was performed as described in the Methods section. The detailed results of this analysis are shown in the Supplementary Figure S4 and Supplementary Table S3. Importantly, none of the viruses, which were previously shown to be neutralized by either of the two tested here mAbs in experimental assays conducted by Hioe et al [11], were predicted as resistant by MDE. This further indicates that the optimized MDE is highly sensitive.

6. Self- and cross-docking validation of the Flexible Peptide Docking (FPD) protocol

Although the general docking method we used has been previously tested under a variety of self- and cross- docking scenarios with excellent accuracy [12,13] (including blind prediction tests [14]), the accuracy is always dependent critically on the quality of the specific receptor crystal structure used in any experiment. Accordingly, we performed multiple self- and cross-docking experiments to control for unforeseen inaccuracies. Specifically, we performed three self- and six cross-docking experiments of the three different V3 peptides (MN, UG1033, UR29) into the three available conformations of mAb 2219 (2B0S, 2B1A, 2B1H), and six self- and eighteen cross-docking experiments of the four V3 peptides (MN, UG1033, 9 amino acid W2RW020, and 10 amino acid W2RW020) into six available confirmations of mAb 447 (1Q1Jp, 1Q1Jq, 3C2Ap, 3C2Aq, 3GHBp, 3GHBq; see Supplementary Table 1). In each experiment, FPD was independently conducted 10 times and average root-mean-square deviation (RMSD) value was calculated between the FPD-predicted structure of a peptide backbone and the crystallographic structure of the same peptide backbone. Small RMSD values mean

small deviation between the predicted and the experimentally obtained structures suggesting that the FPD is accurate.

The results of the analysis are summarized on the Supplementary Figure S5. FPD correctly predicted the correct binding mode and orientation of V3 peptides within the antibody, including the major contact areas in every case. Average RMSD values across all self-dockings (3.08 for 2219 and 2.55 for 447-52D) and all cross-dockings (3.37 for 2219 and 3.07 for 447-52D) are typical for correctly docked peptides where side chains and one or both extreme termini vary, which is the expected variance for this resolution of crystallography.

Specific numbers for different docking experiments (see Supplementary Fig. S5a,b) suggest that FPD works for some V3 sequences better than for others. One such example: RMSD values for the peptide UG1033 docked into different mAb 447-52D conformations are substantially higher than those for the other three peptides (MN, 9 amino acid W2RW020, and 10 amino acid W2RW020). This, however, could be explained by the difference in the peptide length, which is equal to 13 amino acids for UG1033, while 9 or 10 for the other peptides. Indeed, it is much more difficult to predict a longer peptide than a shorter one due to the larger number of free variables. Noise level also is expected to increase substantially in this type of more complex simulation.

In contrast to the 447-52D docking experiments, all three peptides crystalized with the 2219 have the same length of 16 amino acids, but RMSD variation patterns between them still exists (Supplementary Fig. S5). To understand this variation we performed a visual analysis of the predicted V3 peptide structures (Supplementary Fig. S6). This analysis suggests that while there is little deviation in N-terminus end of the V3 peptide, the main structural difference between the experimentally defined crystallographic structure and the FPD-predicted structure is in its C-terminus part. This part of the V3 loop, however, has very few atomic contacts with the mAb comparing to the N-terminal face of the peptide, where the majority of the crucial contacts occur [5]. Also, it may be important that the C-terminal portions of the experimentally obtained UR29 and UG1033 peptide structures form an extended ß-sheet with their cognate crystallographically neighboring Fabs in 2B1A and 2B1H, which is not a case for the MN peptide in 2B0S [5]. Such crystallographic artifacts in the C-terminal structures of UR29 and UG1033 would be expected to deviate from the docking results, which model the conformation in the non-crystal, physiological environment. These structural observations correlate well with the computational results of our current study: our analysis demonstrates that the optimal docking peptide for the mAb 2219 is restricted solely to the N-terminal part of the V3 peptide, and specifically to the positions 10 through 13. Accordingly, our self- and cross-docking results show excellent structural reconstruction in this region of the V3 (Supplementary Fig. S5 panels c and d), suggesting that our FPD protocol is highly accurate.

SUPPLEMENTARY REFERENCES

1. Almond D, Kimura T, Kong X, Swetnam J, Zolla-Pazner S, et al. (2010) Structural conservation predominates over sequence variability in the crown of HIV type 1's V3 loop. AIDS Res Hum Retroviruses 26: 717-723.
2. Burke V, Williams C, Sukumaran M, Kim SS, Li H, et al. (2009) Structural basis of the cross-reactivity of genetically related human anti-HIV-1 mAbs: implications for design of V3-based immunogens. Structure 17: 1538-1546.
3. Jiang X, Burke V, Totrov M, Williams C, Cardozo T, et al. (2010) Conserved structural elements in the V3 crown of HIV-1 gp120. Nat Struct Mol Biol 17: 955-961.
4. Stanfield RL, Gorny MK, Williams C, Zolla-Pazner S, Wilson IA (2004) Structural rationale for the broad neutralization of HIV-1 by human monoclonal antibody 447-52D. Structure 12: 193-204.
5. Stanfield RL, Gorny MK, Zolla-Pazner S, Wilson IA (2006) Crystal structures of human immunodeficiency virus type 1 (HIV-1) neutralizing antibody 2219 in complex with three different V3 peptides reveal a new binding mode for HIV-1 cross-reactivity. J Virol 80: 6093-6105.
6. Matthews BW (1975) Comparison of the predicted and observed secondary structure of T4 phage lysozyme. Biochim Biophys Acta 405: 442-451.
7. Kumar R, Indrayan A (2011) Receiver operating characteristic (ROC) curve for medical researchers. Indian Pediatr 48: 277-287.
8. Cardozo T, Swetnam J, Pinter A, Krachmarov C, Nadas A, et al. (2009) Worldwide distribution of HIV type 1 epitopes recognized by human anti-V3 monoclonal antibodies. AIDS Res Hum Retroviruses 25: 441-450.
9. Swetnam J, Shmelkov E, Zolla-Pazner S, Cardozo T (2010) Comparative Magnitude of Cross-Strain Conservation of HIV Variable Loop Neutralization Epitopes. PLoS One 5: e15994.
10. Hemelaar J, Gouws E, Ghys PD, Osmanov S (2011) Global trends in molecular epidemiology of HIV-1 during 2000-2007. AIDS 25: 679-689.
11. Hioe CE, Wrin T, Seaman MS, Yu X, Wood B, et al. (2010) Anti-V3 monoclonal antibodies display broad neutralizing activities against multiple HIV-1 subtypes. PLoS One 5: e10254.
12. Bordner AJ, Abagyan R (2006) Ab initio prediction of peptide-MHC binding geometry for diverse class I MHC allotypes. Proteins 63: 512-526.
13. Neves MA, Totrov M, Abagyan R (2012) Docking and scoring with ICM: the benchmarking results and strategies for improvement. Journal of computer-aided molecular design 26: 675-686.
14. Fernandez-Recio J, Abagyan R, Totrov M (2005) Improving CAPRI predictions: optimized desolvation for rigid-body docking. Proteins 60: 308-313.

mAb (Docking Model)	2219 (mAb conformation: 2B0S, peptide: 10-13)										447-52D (mAb conformation: 1Q1Jq, peptide: 9-20)									
Simulation #	1	2	3	4	5	6	7	8	9	10	1	2	3	4	5	6	7	8	9	10
Consensus A1	-35.605	-44.228	-39.626	-41.491	-36.920	-37.570	-36.330	-41.580	-38.382	-43.221	-62.056	-69.492	-64.118	-53.967	-69.676	-58.173	-70.780	-65.677	-63.235	-64.469
Consensus AE	-19.984	-19.422	-23.280	-17.962	-21.273	-22.687	-19.478	-22.187	-23.028	-19.295	-63.481	-60.615	-61.766	-51.409	-52.558	-61.494	-60.632	-57.022	-54.790	-55.731
Consensus B	-37.533	-36.907	-37.426	-38.174	-36.019	-36.400	-36.178	-36.572	-34.986	-37.231	-69.386	-70.780	-71.063	-72.584	-67.818	-70.618	-71.779	-69.836	-68.797	-69.845
Consensus B-D29A	-37.533	-36.907	-37.426	-38.174	-36.019	-36.400	-36.178	-36.572	-34.986	-37.231	-69.386	-70.780	-71.063	-72.584	-67.818	-70.618	-71.779	-69.836	-68.797	-69.845
Consensus B-D29N	-37.533	-36.907	-37.426	-38.174	-36.019	-36.400	-36.178	-36.572	-34.986	-37.231	-69.386	-70.780	-71.063	-72.584	-67.818	-70.618	-71.779	-69.836	-68.797	-69.845
Consensus B-H13P	-33.233	-32.297	-33.500	-34.216	-33.121	-32.894	-32.967	-32.829	-33.976	-32.804	-66.552	-62.749	-68.582	-67.058	-70.486	-54.672	-69.298	-67.907	-54.784	-67.841
Consensus B-H13P-I14M	-33.233	-32.297	-33.500	-34.216	-33.121	-32.894	-32.967	-32.829	-33.976	-32.804	-55.270	-61.539	-61.837	-57.999	-55.936	-56.492	-50.847	-55.646	-61.434	-55.913
Consensus B-H13R-T22A-E25D	-35.605	-44.228	-39.626	-41.491	-36.920	-37.570	-36.330	-41.580	-38.382	-43.221	-70.574	-69.796	-70.792	-72.515	-72.916	-73.334	-67.575	-69.831	-72.197	-71.834
Consensus B-H13T-R18Q-A19T-T22A-E25D	-30.330	-33.275	-30.913	-31.502	-31.228	-31.423	-31.906	-31.106	-31.998	-32.360	-53.612	-62.027	-53.324	-57.338	-47.685	-61.056	-55.481	-50.334	-58.259	-64.635
Consensus B-H13T-T22A-E25D	-30.330	-33.275	-30.913	-31.502	-31.228	-31.423	-31.906	-31.106	-31.998	-32.360	-65.852	-65.972	-68.128	-70.267	-68.095	-66.824	-63.368	-65.587	-67.998	-72.706
Consensus B-I12M	-35.848	-35.524	-36.959	-33.658	-34.603	-34.880	-29.892	-36.712	-33.002	-34.987	-65.215	-67.838	-62.389	-69.342	-69.899	-68.158	-72.298	-61.026	-67.331	-66.359
Consensus B-I12M-H13R	-32.243	-32.355	-33.611	-36.749	-32.054	-34.379	-37.683	-33.213	-35.436	-34.040	-64.758	-74.809	-58.328	-72.089	-72.656	-67.339	-71.320	-63.926	-69.364	-68.949
Consensus B-I12V	-36.771	-36.410	-36.525	-37.580	-37.746	-36.208	-37.411	-37.112	-36.683	-37.367	-70.906	-70.874	-66.189	-74.843	-66.989	-66.043	-70.293	-70.320	-67.722	-71.599
Consensus B-I12V-H13R-T22A-E25D	-40.508	-40.951	-45.701	-40.983	-44.106	-39.630	-38.190	-38.734	-43.984	-39.582	-72.283	-71.852	-73.241	-71.687	-70.337	-70.836	-71.417	-69.660	-75.699	-73.225
Consensus B-I14L	-37.533	-36.907	-37.426	-38.174	-36.019	-36.400	-36.178	-36.572	-34.986	-37.231	-68.924	-69.138	-70.530	-68.815	-70.037	-70.007	-69.816	-70.651	-69.792	-71.304
Consensus B-I14M	-37.533	-36.907	-37.426	-38.174	-36.019	-36.400	-36.178	-36.572	-34.986	-37.231	-70.220	-74.916	-63.166	-70.696	-69.525	-70.325	-67.600	-69.763	-70.221	-70.467
Consensus B-K10A	-26.585	-26.106	-26.354	-26.087	-26.246	-26.616	-26.500	-26.687	-26.495	-26.945	-69.680	-66.704	-68.741	-60.892	-68.510	-69.057	-70.268	-68.733	-64.501	-67.839
Consensus B-K10E	-27.012	-26.455	-25.856	-25.721	-26.447	-27.282	-25.803	-26.793	-25.690	-27.347	-67.913	-67.712	-68.510	-68.254	-62.895	-57.971	-61.240	-67.897	-67.851	-67.934
Consensus B-P16A	-37.533	-36.907	-37.426	-38.174	-36.019	-36.400	-36.178	-36.572	-34.986	-37.231	-67.762	-67.075	-66.015	-62.828	-66.756	-72.004	-59.052	-73.888	-71.072	-66.056
Consensus B-R18K	-37.533	-36.907	-37.426	-38.174	-36.019	-36.400	-36.178	-36.572	-34.986	-37.231	-61.236	-59.266	-69.059	-63.337	-65.991	-65.467	-68.972	-64.746	-71.004	-66.965
Consensus B-R18Q	-37.533	-36.907	-37.426	-38.174	-36.019	-36.400	-36.178	-36.572	-34.986	-37.231	-68.388	-60.163	-62.273	-57.753	-60.829	-65.493	-65.971	-59.824	-65.760	-63.299
Consensus B-R3A	-37.533	-36.907	-37.426	-38.174	-36.019	-36.400	-36.178	-36.572	-34.986	-37.231	-69.386	-70.780	-71.063	-72.584	-67.818	-70.618	-71.779	-69.836	-68.797	-69.845
Consensus B-R3Q	-37.533	-36.907	-37.426	-38.174	-36.019	-36.400	-36.178	-36.572	-34.986	-37.231	-69.386	-70.780	-71.063	-72.584	-67.818	-70.618	-71.779	-69.836	-68.797	-69.845
Consensus B-R9A	-37.533	-36.907	-37.426	-38.174	-36.019	-36.400	-36.178	-36.572	-34.986	-37.231	-68.132	-62.070	-63.874	-65.619	-49.436	-61.451	-69.042	-64.786	-63.702	-62.881
Consensus B-R9E	-37.533	-36.907	-37.426	-38.174	-36.019	-36.400	-36.178	-36.572	-34.986	-37.231	-61.659	-66.078	-62.181	-67.560	-68.601	-63.707	-64.627	-62.509	-59.943	-63.347
Consensus B-T22A	-37.533	-36.907	-37.426	-38.174	-36.019	-36.400	-36.178	-36.572	-34.986	-37.231	-69.386	-70.780	-71.063	-72.584	-67.818	-70.618	-71.779	-69.836	-68.797	-69.845
Consensus B-T22A-E25D	-37.533	-36.907	-37.426	-38.174	-36.019	-36.400	-36.178	-36.572	-34.986	-37.231	-69.386	-70.780	-71.063	-72.584	-67.818	-70.618	-71.779	-69.836	-68.797	-69.845
Consensus B-T22A-Q32K	-37.533	-36.907	-37.426	-38.174	-36.019	-36.400	-36.178	-36.572	-34.986	-37.231	-69.386	-70.780	-71.063	-72.584	-67.818	-70.618	-71.779	-69.836	-68.797	-69.845
Consensus B-T8I	-37.533	-36.907	-37.426	-38.174	-36.019	-36.400	-36.178	-36.572	-34.986	-37.231	-69.386	-70.780	-71.063	-72.584	-67.818	-70.618	-71.779	-69.836	-68.797	-69.845
Consensus C	-35.605	-44.228	-39.626	-41.491	-36.920	-37.570	-36.330	-41.580	-38.382	-43.221	-61.582	-65.681	-66.872	-63.367	-62.339	-62.419	-63.203	-62.472	-62.941	-66.706
Consensus C-D25E	-35.605	-44.228	-39.626	-41.491	-36.920	-37.570	-36.330	-41.580	-38.382	-43.221	-61.582	-65.681	-66.872	-63.367	-62.339	-62.419	-63.203	-62.472	-62.941	-66.706
Consensus C-I12M-D25E	-32.243	-32.355	-33.611	-36.749	-32.054	-34.379	-37.683	-33.213	-35.436	-34.040	-70.757	-65.459	-64.410	-59.670	-70.614	-68.385	-62.154	-63.995	-65.300	-57.571
Consensus C-I12M-R13P-T19A-D25E	-32.195	-30.321	-31.039	-28.863	-29.526	-30.816	-31.351	-31.003	-29.834	-31.743	-56.110	-55.955	-58.370	-45.702	-55.695	-54.663	-53.372	-53.842	-58.083	-55.329
Consensus C-I12M-R13S-T19A-D25E	-29.054	-31.731	-29.443	-29.886	-30.548	-32.598	-32.137	-31.486	-26.354	-30.365	-59.104	-52.354	-54.780	-51.621	-56.352	-57.983	-48.693	-50.792	-53.775	-55.516
Consensus C-I12V	-40.508	-40.951	-45.701	-40.983	-44.106	-39.630	-38.190	-38.734	-43.984	-39.582	-68.685	-62.183	-72.842	-69.201	-63.338	-64.328	-65.082	-64.337	-63.659	-64.749
Consensus C-I14F-D25E	-35.605	-44.228	-39.626	-41.491	-36.920	-37.570	-36.330	-41.580	-38.382	-43.221	-75.657	-64.295	-63.397	-60.872	-60.987	-58.839	-65.246	-57.471	-58.573	-63.042
Consensus C-I14L-D25E	-35.605	-44.228	-39.626	-41.491	-36.920	-37.570	-36.330	-41.580	-38.382	-43.221	-64.482	-65.675	-60.068	-59.767	-64.208	-75.735	-70.004	-63.169	-63.271	-63.192
Consensus C-I14M-D25E	-35.605	-44.228	-39.626	-41.491	-36.920	-37.570	-36.330	-41.580	-38.382	-43.221	-72.433	-63.762	-69.985	-65.738	-63.500	-66.539	-59.120	-66.322	-64.007	-64.370
Consensus C-I14V-D25E	-35.605	-44.228	-39.626	-41.491	-36.920	-37.570	-36.330	-41.580	-38.382	-43.221	-64.759	-58.676	-70.662	-71.903	-64.281	-69.339	-60.917	-59.550	-60.295	-60.718
Consensus C-K10A-D25E	-26.192	-26.011	-29.293	-26.470	-27.847	-28.369	-26.273	-25.294	-27.324	-30.278	-61.888	-62.000	-61.666	-63.331	-61.553	-67.664	-64.246	-60.192	-64.484	-60.270
Consensus C-K10A-I14M-D25E	-26.192	-26.011	-29.293	-26.470	-27.847	-28.369	-26.273	-25.294	-27.324	-30.278	-68.951	-68.609	-64.652	-62.541	-67.919	-67.119	-69.009	-62.158	-62.132	-62.187
Consensus C-K10E-D25E	-30.080	-29.705	-27.092	-28.514	-26.789	-31.550	-31.868	-31.836	-25.343	-28.546	-67.592	-61.964	-61.373	-71.770	-60.098	-60.678	-62.581	-58.946	-55.880	-67.345
Consensus C-K10R-R13P-I14V-D25E	-32.159	-28.855	-29.020	-29.179	-30.158	-30.126	-27.538	-26.434	-28.874	-26.395	-51.466	-61.158	-66.366	-51.464	-48.757	-52.205	-63.299	-63.383	-62.562	-66.086
Consensus C-R13N-I14F-T19A	-34.756	-35.371	-35.555	-36.132	-35.769	-34.991	-34.192	-35.022	-35.501	-35.547	-55.591	-56.567	-55.845	-53.519	-59.483	-61.313	-53.467	-55.234	-66.066	-65.018
Consensus C-R13P-T19A-D25E	-33.233	-32.297	-33.500	-34.216	-33.121	-32.894	-32.967	-32.829	-33.976	-32.804	-54.175	-62.074	-53.524	-50.938	-55.592	-56.303	-59.163	-51.294	-53.253	-56.672
Consensus C-R13S-T19A-D25E	-30.583	-31.121	-30.778	-31.517	-32.613	-32.471	-31.149	-31.954	-29.829	-31.458	-59.544	-59.160	-60.109	-47.775	-64.857	-48.052	-57.499	-65.046	-56.562	-58.037
Consensus C-R13T-I14F-T19A-D25E	-30.330	-33.275	-30.913	-31.502	-31.228	-31.423	-31.906	-31.106	-31.998	-32.360	-48.721	-58.086	-46.238	-53.402	-53.658	-50.612	-51.843	-65.062	-57.710	-52.020
Consensus C-R13T-I14L-T19A-D25E	-30.330	-33.275	-30.913	-31.502	-31.228	-31.423	-31.906	-31.106	-31.998	-32.360	-53.038	-60.159	-59.751	-53.034	-48.296	-62.070	-53.426	-70.323	-57.638	-60.160
Consensus C-R9A-D25E	-35.605	-44.228	-39.626	-41.491	-36.920	-37.570	-36.330	-41.580	-38.382	-43.221	-63.515	-52.839	-58.908	-56.765	-60.253	-56.701	-56.313	-52.525	-53.215	-57.985
Consensus C-R9A-I14V-D25E	-35.605	-44.228	-39.626	-41.491	-36.920	-37.570	-36.330	-41.580	-38.382	-43.221	-60.050	-66.133	-54.686	-57.862	-62.805	-54.288	-55.189	-62.626	-59.010	-57.353
Consensus C-R9A-R13G-D25E	-24.055	-28.546	-26.348	-23.956	-25.496	-23.768	-25.193	-23.171	-26.402	-26.385	-55.281	-57.191	-52.642	-59.406	-56.826	-60.444	-54.331	-53.904	-50.409	-56.967
Consensus C-R9E-D25E	-35.605	-44.228	-39.626	-41.491	-36.920	-37.570	-36.330	-41.580	-38.382	-43.221	-58.966	-66.753	-53.815	-55.699	-57.208	-55.191	-50.319	-53.049	-57.747	-58.402
Consensus C-R9I-I12M-R13G-D25E	-24.661	-25.006	-26.081	-27.788	-24.868	-25.806	-27.332	-25.531	-24.300	-25.245	-51.275	-48.595	-57.505	-58.651	-50.289	-53.888	-62.963	-54.668	-51.816	-55.775
Consensus C-R9I-K10Q-I12M-R13G-D25E	-27.659	-27.698	-27.483	-28.189	-27.471	-27.585	-27.618	-27.347	-27.942	-27.656	-47.517	-46.650	-50.690	-50.105	-49.800	-49.695	-55.514	-54.240	-56.234	-43.006
Consensus C-R9Q-I14V-D25E	-35.605	-44.228	-39.626	-41.491	-36.920	-37.570	-36.330	-41.580	-38.382	-43.221	-68.391	-65.918	-58.176	-59.358	-61.253	-63.931	-65.468	-65.319	-54.723	-63.269
Consensus C-R9T-I14V-D25E	-35.605	-44.228	-39.626	-41.491	-36.920	-37.570	-36.330	-41.580	-38.382	-43.221	-58.757	-57.679	-57.824	-65.603	-61.244	-57.328	-61.341	-69.809	-60.100	-57.370
Consensus C-S11G-R13G-D25E	-30.144	-24.355	-26.276	-25.795	-25.572	-25.054	-24.038	-28.959	-25.852	-25.158	-59.663	-66.997	-63.324	-59.121	-53.577	-53.827	-59.499	-52.647	-58.441	-60.688
Consensus F	-37.533	-36.907	-37.426	-38.174	-36.019	-36.400	-36.178	-36.572	-34.986	-37.231	-68.388	-60.163	-62.273	-57.753	-60.829	-65.493	-65.971	-59.824	-65.760	-63.299
Consensus H	-37.533	-36.907	-37.426	-38.174	-36.019	-36.400	-36.178	-36.572	-34.986	-37.231	-59.104	-70.443	-63.825	-65.026	-62.041	-64.276	-63.986	-60.786	-64.660	-56.884

Supplementary Figure S7: **Predicted binding energies of V3 peptides to mAbs 2219 and 447-52D.** For each V3 peptide representing the experimental set of 59 psVs, binding energies were calculated using the preselected optimal docking models as described in the Methods section. The results of the ten independent simulations are shown.
